# Supplementary material for: Controlling crystallization in covalent organic frameworks to facilitate photocatalytic hydrogen production
Source: Nat Commun. 2025 Feb 24;16:1940. doi: 10.1038/s41467-025-57166-1 (PMC11850636; doi:10.1038/s41467-025-57166-1)
Supplement: Supplementary file 1 — Supplementary Information [file 41467_2025_57166_MOESM1_ESM.pdf]

Supplementary Information

Controlling crystallization in covalent organic frameworks to facilitate photocatalytic hydrogen production

Zheng Lin[1,5], Xiangkun Yu[2,5], Zijian Zhao[3], Ning Ding[1], Changchun Wang[1], Ke Hu[3,4*], Youliang Zhu[2*], Jia Guo[1*]

[1]*State Key Laboratory of Molecular Engineering of Polymers, Department of Macromolecular Science, Fudan University, Shanghai 200438, China*

[2]*State Key Laboratory of Supramolecular Structure and Materials, College of Chemistry, Jilin University, Changchun 130023, China*

[3]*Department of Chemistry, Fudan University, Shanghai 200438, China*

[4]*School of Chemical Science and Engineering, Tongji University, Shanghai 200092, China*

[5]*These authors contributed equally: Zheng Lin, Xiangkun Yu*

**E-mail: khu5@alumni.jh.edu (K. H.), youliangzhu@jlu.edu.cn (Y. Z.), guojia@fudan.edu.cn (J. G.)*

Section I. Material and Methods

1. Materials

Anhydrous mesitylene, 1,4-dioxane, aniline, acetic acid, ascorbic acid, and chloroplatinic acid hexahydrate were purchased from Aladdin Industrial Corporation. 1,3,5-triformylphloroglucinol (Tp) was purchased from Jilin Chinese Academy of Sciences-Yanshen technology Co. Ltd. *p*-Phenylenediamine (Pa), benzidine (BD) was purchased from TCI Shanghai. 2,5-diaminopyridine (Py) was purchased from Macklin Inc. Tetrahydrofuran (THF) and ethanol were purchased from Sinopharm Chemical Reagent Co. Ltd. 5% Nafion solution was purchased from DuPont Company. All the chemical materials were used without further purification.

2. Characterizations

Powder X-ray diffraction (PXRD) patterns were collected at room temperature on an X-ray diffraction spectrometer (Bruker D8 Advance, Germany) with Cu Kα radiation at $\lambda = 0.154$ nm operating at 40 kV and 40 mA. Fourier transform infrared (FT-IR) spectra were recorded on Nicolet 6700 (Thermofsher, USA) Fourier transformation infrared spectrometer. UV-vis absorption spectra were collected on a Lambda 750 spectrometer (referenced to barium sulphate). The photoluminescent spectra were recorded on the Edinburgh FLS1000 photoluminescence spectrometer. Transmission electron microscopy images were obtained using a Tecnai G2 20 TWIN microscope operated at 200 kV accelerating voltage. High-resolution transmission electron microscopy images were obtained using a Tecnai G2 F20 S-Twin microscope operated at 200 kV accelerating voltage. N_2 adsorption-desorption isotherms were collected by a TriStar II 3020 volumetric adsorption analyzer (Micromeritics, USA) at 77 K. The samples were degassed at 120 °C for 12 h under vacuum before measurement. The surface areas were evaluated using the Brunauer-Emmett-Teller (BET) model applied between P/P_0 values of 0.05 and 0.1 for COFs. Solid-state CP/MAS ^{13}C NMR spectra were recorded on 400WB AVANCE III (Bruker, Switzerland) plus 400 MHz spectrophotometer at 298 K. The platinum content of materials after photo-deposition

was tested by inductively coupled plasma atomic emission spectroscopy (ICP-AES) using the Agilent ICPMS 7700 and Agilent 720ES spectrometer. X-ray photoelectron spectroscopy (XPS) measurements were analyzed using a Thermo Scientific K-alpha XPS instrument equipped with an Al K source (1486.6 eV). Thermogravimetric analysis (TGA) was conducted on a Pyris 1 Thermo Gravimetric Analyzer (PE, USA) at a heating rate of 10°C/min from 100°C to 800°C under air atmosphere. The Si element contents were measured by inductively coupled plasma atomic emission spectroscopy (ICP-AES) using Aligent720ES spectrometer. The zeta potentials were measured by a dynamic light scattering (DLS) particle size analyzer (Malvern Nano-ZS90, UK) at a scattering angle of 90°. Each sample was tested for three times and averaged. The surface potential distributions were characterized on Bruker Dimension Icon using KPFM mode. The surface photovoltage spectra were acquired on a Surface photovoltage spectrometer (CEL-SPS1000).

A TSP-2000 (Unisoku) laser flash photolysis system was employed to record nanosecond transient absorption spectra. Briefly, a Q-switched frequency-doubled Nd:YAG laser (Quantel Q-Smart 450, 10 Hz), along with an OPO (MagicPRISM VIS, OPOtek), served as the excitation light source. The laser pulse wavelength was tuned at 532 nm (with a full width at half maximum of 5-8 ns and an energy density of 5 mJ/cm^2 per pulse). A 75 W xenon lamp was utilized as the detection beam, which was focused onto the sample in a 10 mm cuvette. The detection beam was arranged perpendicular to the excitation laser pulses and directed into a f/4 monochromator (Acton, Princeton Instrument) equipped with a photomultiplier tube (R2949, Hamamatsu) for single wavelength detection. Simultaneous full spectrum detection at each time delay was achieved with an iCCD camera (iStar 320T, Andor). Transient absorption data at each wavelength were recorded using a digital oscilloscope (LeCroy 4024, 12-bit, 200 MHz) connected to a computer, typically averaging 50-100 laser pulses. Single wavelength kinetic data were digitized on a logarithmic time scale. All sample solutions were purged with N_2 or Ar for 15 minutes prior to the transient

absorption experiments. The absorbances of the samples were adjusted to 0.4-0.8 at the indicated excitation wavelength.

3. Methods

3.1 Synthesis of TpPa-SCOF

The synthesis of TpPa-SCOF was carried out using the modified two-step method.[1] In the first step, a template-free precipitation polymerization was carried out to prepare the TpPa-Polymer-An. Typically, Tp (14.0 mg, 0.067 mmol) and Pa (10.8 mg, 0.1 mmol) were dissolved in 5 mL anhydrous ethanol, respectively, to get clear solutions. Then the two monomer solutions were rapidly mixed and heated to reflux under magnetic stirring. After 15-h reaction, the solvent was removed by rotary evaporation to obtain the dark orange product. In the second step, the TpPa-Polymer microspheres were treated in the typical solvothermal conditions. The dark orange solids were transferred into a Pyrex tube (10 mL) with the mixed solvent of mesitylene and dioxane (1/1 by *vol.*; 2 mL) and 6M HOAc (0.2 mL). After three freeze-pump-thaw cycles, the tube was sealed and kept at 120°C in an oven for 3 days. The products were collected by centrifugation, washed with THF for several times and dried at 40°C under vacuum to give the TpPa-SCOF powder with a yield of 91%.

3.2 Synthesis of TpPa-COF[2]

A Pyrex tube (10 mL) was charged with Tp (21.0 mg, 0.1 mmol), Pa (16.2 mg, 0.15 mmol), mesitylene (0.5 mL), dioxane (0.5 mL) and 6M HOAc (0.1 mL). The mixture was sonicated for 2 min and then degassed through three freeze-pump-thaw cycles before sealing under vacuum. The sealed tube was kept at 120°C in an oven for 3 days. The precipitate was collected by filtration, washed with THF for several times. Then the product was Soxhlet extracted with THF for 1 day and dried under vacuum at 40°C for 24 h to collect TpPa-COF powder with a yield of 87%.

3.3 Synthesis of TpPa-COF-An

A Pyrex tube (10 mL) was charged with Tp (14.0 mg, 0.067 mmol), Pa (10.8 mg, 0.1 mmol), aniline (0.1 mL, 16 *equiv.* relative to the amount of Tp), mesitylene (0.5 mL), dioxane (0.5 mL) and 6M HOAc (0.1 mL). The mixture was sonicated for 2 min and then degassed through three freeze-pump-thaw cycles before sealing under vacuum. The sealed tube was kept at 120°C in an oven for 3 days. The precipitate was collected by filtration, washed with THF for several times. Then the product was Soxhlet extracted with THF for 1 day and dried under vacuum at 40°C for 24 h to collect TpPa-COF-An powder with a yield of 85%.

3.4 Synthesis of TpPy-COF and TpPy-SCOF

The synthesis recipes for TpPy-COF, TpPy-COF-An, TpPy-SCOF, and TpPy-SCOF-An were similar to those for TpPa-COF, TpPa-COF-An, TpPa-SCOF, and TpPa-SCOF-An, except that the linker Pa (10.8 mg) was replaced by Py (10.8 mg).

3.5 Synthesis of TpBD-COF and TpBD-SCOF

The synthesis recipes for TpBD-COF, TpBD-COF-An, TpBD-SCOF, and TpBD-SCOF-An were similar to those for TpPa-COF, TpPa-COF-An, TpPa-SCOF, and TpPa-SCOF-An, except that the linker Pa (10.8 mg) was replaced by BD (18.4 mg).

3.6 Synthesis of SiO_2

The SiO_2 microsphere was prepared through a modified Stöber method. Specifically, 5.55 mL TEOS was added into 87 mL arginine aqueous solution (1 mg/mL) and the system was heated to 70°C under magnetic stirring for 24 h. After cooling to room temperature, the solution was collected as a seed stock solution. Next, 200 mL anhydrous ethanol, 14 mL deionized water, and 8 mL ammonia water were mixed and added with 200 μL of the seed stock solution. After that, 14.95 g TEOS was continuously added to the system with a feeding rate of 2 mL/h. After the TEOS was added, the reaction was allowed for 6 h to ensure complete polymerization. The

products were collected by centrifugation and washed several times with deionized water and ethanol.

3.7 Synthesis of SiO_2@TpPa-An

The SiO_2@TpPa-An microspheres were prepared by using the template-mediated two-step method. In the first step, a template-mediated precipitation polymerization was conducted to prepare the core-shell microspheres consisting of SiO_2 in core and amorphous polymer network in shell. Typically, the dispersion of SiO_2 (25 mg) in THF (12.5 mL) was mixed with Tp (14.0 mg) under ultra-sonication for 10 min, resulting in a homogenous solution. The obtained solution heated to reflux under magnetic stirring allowing for the interaction between –CHO of SiO_2 and $–NH_2$ groups on the SiO_2 surface. After 30 min, a THF solution of Pa (10.8 mg, 2.5 mL) was added to the above dispersion at a feeding rate of 0.18 mL/min, and the reaction was allowed to proceed for 12 h. The solvent was removed by rotary evaporation to obtain the orange product. In the second step, the SiO_2@Polyimine microspheres were treated by the typical solvothermal method. The orange solids were transferred into a Pyrex tube (10 mL) with the mixed solvent of mesitylene/dioxane (1/1 by *vol.*; 2 mL) and 6M HOAc (0.2 mL). After three freeze-pump-thaw cycles, the tube was sealed and kept at 120°C in an oven for 3 days. The products were collected by centrifugation, washed with THF for several times and dried at 40°C under vacuum to give the TpPa-SCOF-An as red powder with the yield of 85%.

3.8 Simulation details

Due to the large time and length scales in experimental system, we performed molecular dynamics (MD) simulations with coarse-grained (CG) models, which take a group of atoms as a bead (Fig. 1a). Specifically, Tp was represented by a bead-spring model with one monomeric unit as two beads (type A and type B). Pa as linker molecules are implicitly included in polymerization. In each reaction event, a B-B bond along with two A-B-B angles are generated. The spherical COFs with a radius of 19 nm consisting of 2,588 monomers (10, 352 beads) and the bulk phase consisting of 2,550 monomers

(10, 200 beads) in a 100 nm × 100 nm × 3 nm box under periodic boundary condition were simulated by Brownian dynamics under a constant volume and temperature (NVT) ensemble with T* = 1.0. The simulations were performed for t = 10^5 τ with an integration time step dt = 0.001τ. All simulations were performed with reduced units, with the energy, the bead diameter, and the bead mass in units of ε, σ, m, respectively. Therefore, the time has a unit of $\tau = \sigma\sqrt{m/\varepsilon}$.

The interactions between all CG beads in the system are depicted by non-bonded and bonded interaction terms. The non-bonded potential involves short-range Lennard-Jones (LJ) interaction, which is described as,

$$\varphi_{\text{nb}}(r) = \begin{cases} 4\varepsilon\left[\left(\frac{\sigma}{r}\right)^{12} - \left(\frac{\sigma}{r}\right)^{6}\right] + \varepsilon, & r < \mathrm{r_{cut}} \\ 0, & r \geq \mathrm{r_{cut}} \end{cases}$$

where ε is the interaction strength between different beads, $\mathrm{r_{cut}}$ is the cut-off distance. For beads in the same layer, we employed Weeks-Chandler-Andersen (WCA) repulsive potential with $\mathrm{r_{cut}} = 2^{1/6}\sigma$, $\varepsilon = 1.0$ and $\sigma = 1.0$. For beads in the adjacent layers, we employed LJ potential with $\mathrm{r_{cut}} = 1.5\sigma, \varepsilon = 2.0$ and $\sigma = 1.0$. The bonded potentials describing the interactions of bond stretching and angle bending are given by harmonic form,

$$\varphi_{\text{bond}}(r) = \mathrm{K_{bond}}(r - \mathrm{r_0})^2$$

$$\varphi_{\text{angle}}(\theta) = \mathrm{K_{angle}}(\theta - \theta_0)^2$$

where spring constant $\mathrm{K_{bond}}$ is set as 1250.0 and $\mathrm{K_{angle}}$ is set as 1000.0. The equilibrium length $\mathrm{r_0}$ is 1.0 in reduced units. After the reaction, the generated angles with equilibrium degrees θ_0 are ∠B-A-B = 120° and ∠A-B-B = 180°.

We employed a dynamic bond model to describe the reaction between active beads B by using a set of parameters including a reactive radius of $2^{1/6}$, and a reaction period of 2000 time steps. The interaction and reaction parameters employed here have been calibrated before.[3,4] A forward reaction probability P_f and a reverse reaction probability P_r are expressed as,

$$P_{\mathrm{f}} = \begin{cases} 1, & \varphi_{\mathrm{nb}} \geq E_{\mathrm{bar}} \\ \exp\left(\dfrac{\varphi_{\mathrm{nb}}(r) - E_{\mathrm{bar}}}{\mathrm{k_B T}}\right), & \varphi_{\mathrm{nb}} < E_{\mathrm{bar}} \end{cases}$$

$$P_{\mathrm{r}} = \begin{cases} 1, & \varphi_{\mathrm{bonded}} \geq E_{\mathrm{bar}} + E_{\mathrm{bind}} \\ \exp\left(\dfrac{\varphi_{\mathrm{bonded}}(r,\theta) - E_{\mathrm{bar}} - E_{\mathrm{bind}}}{\mathrm{k_B T}}\right), & \varphi_{\mathrm{bonded}} < E_{\mathrm{bar}} + E_{\mathrm{bind}} \end{cases}$$

where $\varphi_{\mathrm{nb}}(r)$ is the nonboned potential and $\varphi_{\mathrm{bonded}}(r,\theta) = \frac{[\varphi_{\mathrm{bond}}(r) + \sum \varphi_{\mathrm{angle}}(\theta)]}{\mathrm{n_f}}$ is the averaged bonded potential of one B-B bond and two ∠A-B-B bond angles with $\mathrm{n_f} = 3$. All simulations are achieved on GPUs using an in-house package.[5,6]

3.9 The calculation of mean squared displacement (MSD)

MSD is a measure of how far beads move in a system over time and can be calculated as follows,

$$MSD(\tau) = \frac{1}{\mathrm{N}} \sum_{i=1}^{\mathrm{N}} |\mathbf{r}_i(\tau) - \mathbf{r}_i(0)|^2$$

where N is the number of beads, $\mathbf{r}_i(0)$ and $\mathbf{r}_i(\tau)$ is the position of bead *i* at initial time and time τ. In our simulation, it was obtained by tracking the trajectory of beads in the system.

3.10 The AQE measurement

The apparent quantum efficiency (AQE) was measured under the irradiation of a 300W Xe lamp equipped with different bandpass filters (including 475 nm, 550nm, 600 nm, 650 nm and 700 nm) using the following equation.

$$\eta_{AQE} = \frac{2 \times M \times N_A}{\frac{E_{total}}{E_{photon}}} \times 100\% = \frac{2M \times N_A}{\frac{S \times P \times t}{\hbar \times \frac{c}{\lambda}}} \times 100\% = \frac{2 \times M \times N_A \times \hbar \times \mathrm{c}}{S \times P \times t \times \lambda} \times 100\%$$

Where, M is the amount of H_2 molecules (mol), N_A is Avogadro constant (6.022 $\times 10^{23}$ mol^{-1}), $\hbar$ is the Planck constant (6.626 $\times$ 10^{-34} J s), c is the speed of light (3 $\times 10^{8}$ m s^{-1}), S is the irradiation area (cm^2), P is the intensity of irradiation light (W

cm^{-2}), t is the photoreaction time (s), and λ is the wavelength of the monochromatic light (m).

3.11 Photoelectrochemical measurements

Photocurrent response tests, electrochemical impedance spectra (EIS) and Mott-Schottky plots were recorded on the CHI760E electrochemical workstation (Chenhua, China). 5 mg samples dispersed in a mixture of 150 μL ethanol, 350 μL deionized water, and 30 μL 5% Nafion solution was sonicated for 30 min and then the mixture was coated on the indium-tin oxide (ITO) glass to form a film (1 cm^2) as the working electrode. Pt plate was used as the counter electrode, the Ag/AgCl electrode as a reference electrode and 0.5 M Na_2SO_4 as the electrolyte. 300 W Xe lamp equipped with a cut-off filter (>420 nm) was used as light source.

The voltammetry behaviors of the photocatalysts before and after Pt deposition were characterized using linear sweep voltammetry at the scan rate of 5 mV s^{-1}. 0.5 M Na_2SO_4 was applied as the electrolyte. Pt plate was the counter electrode and the Ag/AgCl electrode was the reference electrode. 6 μL dispersion of samples was drop-cast onto the polished glass carbon electrode to form the working electrode. The overpotential was defined as the potential at the current density of 10 mA cm^{-2}.[7]

The applied potentials *vs.* Ag/AgCl are converted to RHE potentials using the following equation,

$$E_{RHE} = E_{Ag/AgCl} + 0.0591pH + E^{\theta}_{Ag/Agcl}(E^{\theta}_{Ag/Agcl} = 0.199\text{V})$$

3.12 Evaluation of built-in electronic field (BEF)

BEF is calculated based on a function of surface potential and surface charge density.[8-11]

$$F = \sqrt{\frac{-2\rho V_s}{\varepsilon_0 \varepsilon_r}}$$

Where F is the BEF magnitude, V_s is the surface potential, ρ is the surface charge density, ε_r is the low-frequency dielectric constant, and ε_0 is the vacuum dielectric constant. So, the BEF intensity is mainly determined by surface voltage and surface charge density while other parameters are constant for TpPa-SCOFs.

The microscopic surface potential V_s of single particle is measured by Kelvin Probe Force Microscopy (KPFM) in the mode of surface potential (**Supplementary Fig. 31**). Specifically, the newly synthesized SCOF sample (1 mg) were dispersed in 10 mL anhydrous ethanol and sonicated with a frequency of 40 kHz at room temperature for 30 min. 10 μL of the dispersion was placed on the surface of the silicon chip and dried at room temperature to remove the solvent for the further KPFM test. For each measurement, a known bias is applied between the sample and the tip of the needle to obtain the surface potential distribution of the sample.

The macroscopic surface potential V_s of sample is acquired by measuring the surface photovoltage spectra (**Supplementary Fig. 32**). The powder sample was sandwiched between two ITO glass. The test system includes a Xe light source, a monochromator, a chopper, and a lock-in amplifier.

The surface charge density ρ is measured by zeta potential and then calculated based on the model proposed by Gouy-Chapman.[8]

$$\sigma = \sqrt{8KT\varepsilon\varepsilon_0 n}\,\sinh\left(\frac{Ze_0\psi_0}{2kT}\right)$$

$$\psi_0 = \zeta(1 + \frac{D}{a_1})e^{\kappa D}$$

where σ is surface charge density (C m^{-2}), k is the Boltzmann constant (J·K^{-1}), T is the absolute temperature (K), n is number of electrolytes per unit volume (m^{-3}), e_0 is electron charge (C), Z represents the electrolyte valence, ψ_0 is the surface potential (V), a_1 is particle stokes radius (m), ζ is zeta potential (mV, Supplementary Fig. 16),

κ^{-1} is Debye length (nm) and D represent the distance from sliding layer to particle surface.

Therefore, the BEF intensity as a function of surface potential V_s and Zeta potential ζ can be simplified as below,

$$\mathrm{F} = (\mathrm{A}V_s \sinh (\frac{Ze_0\zeta\left(1+\frac{D}{a_1}\right)e^{\kappa D}}{2kT}))^{\frac{1}{2}}$$

Section II. Supplementary Figures and Tables

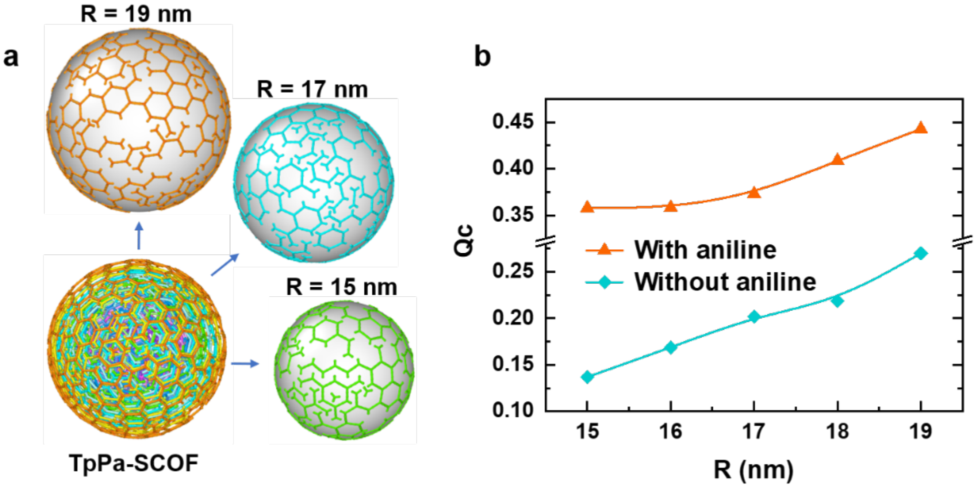

Supplementary Fig. 1 CG-MD Simulation for the spherical COF crystallization with or without aniline.

(a) Snapshots of spherical COFs formed by a two-step process without aniline. (b) Crystallization quality (Qc) for the crystallization of spherical COFs with or without aniline as a function of the radius R of nanospheres. In the simulation, $E_{bind} = 15\ \mathrm{k_B}T$ corresponds to the weak reverse growth of SCOFs without aniline, while $E_{bind} = 9\ \mathrm{k_B}T$ corresponds to the strong reverse growth of SCOFs with suitable amount of aniline. This relationship between E_{bind} and reversibility has been proven in previous work.[5]

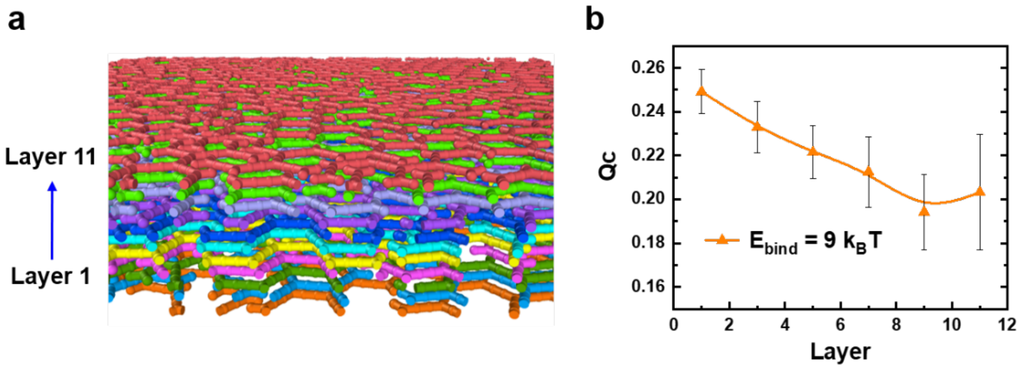

Supplementary Fig. 2 CG-MD simulation for the bulk COF crystallization with aniline.

(a) The snapshot of coarse-grained molecular dynamics simulation of COF crystallization via the bottom-up method with aniline in the bulk phase**.** The different layers of the bulk TpPa-COF-An are marked by different colors. (b) Crystallization quality (Qc) as a function of the position of TpPa-COF-An layers at $E_{\mathrm{bind}} = 9.0\ \mathrm{k_BT}$.

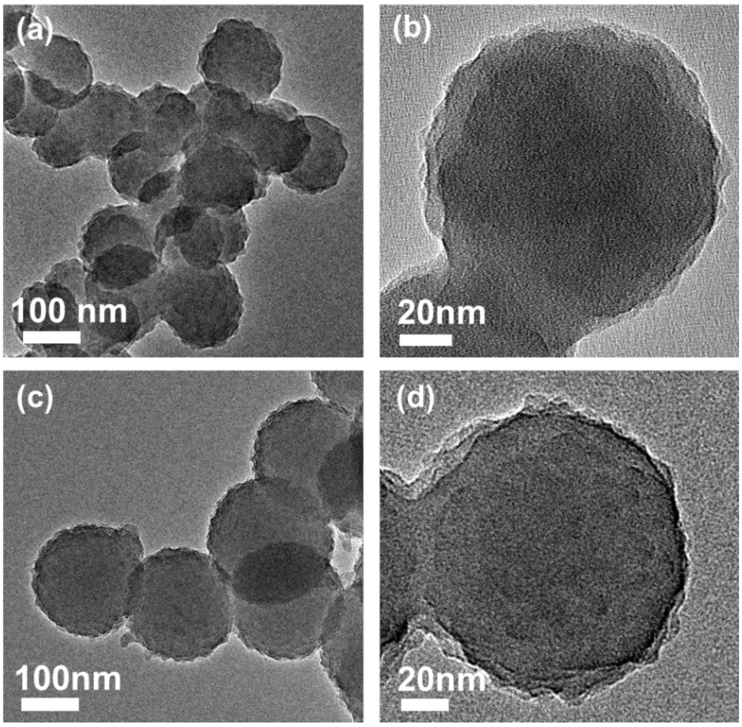

Supplementary Fig. 3 TEM images of spherical TpPa products before and after crystallization.

TEM images of (a,b) TpPa-Polymer and (c,d) TpPa-SCOF.

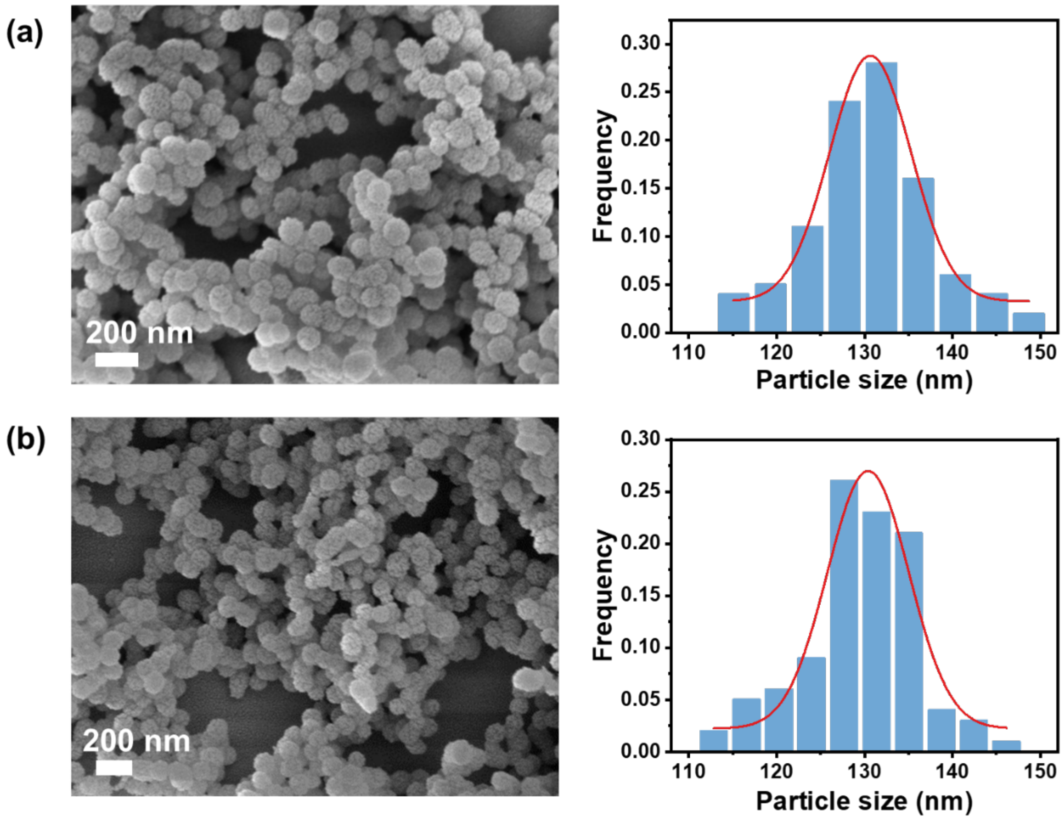

Supplementary Fig. 4 SEM images and particle size distributions of the spherical COFs.

SEM images and particle size distributions for (a) TpPa-SCOF-An and (b) TpPa-SCOF.

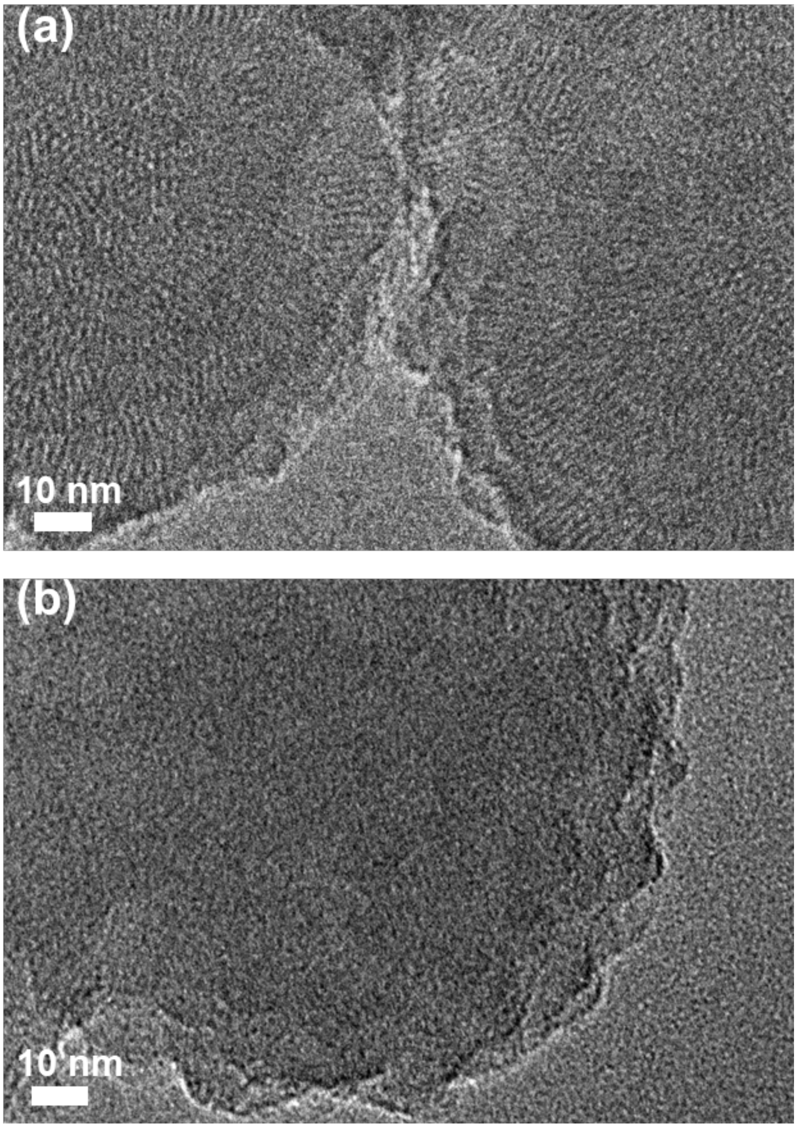

Supplementary Fig. 5 HR TEM images of the spherical COFs.

HR TEM images of the surface crystalline structures for (a) TpPa-SCOF-An and (b) TpPa-SCOF. The comparison shows the improved ordered pore alignment on the surface of TpPa-SCOF-An.

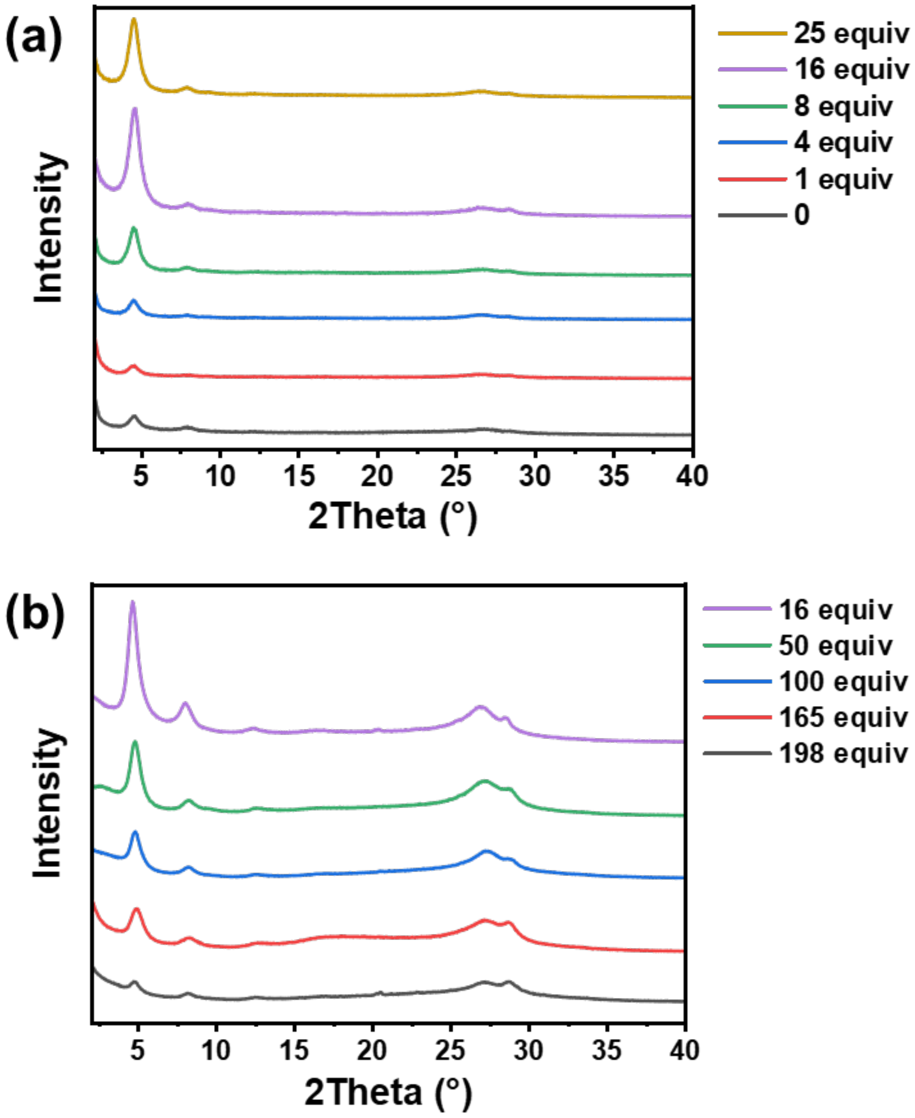

Supplementary Fig. 6 PXRD patterns for TpPa-SCOF-An synthesized under different conditions.

PXRD patterns of the TpPa-SCOF-An prepared by adding (a) 0, 1, 4, 8, 16, and 25 *equiv.* of aniline and (b) 16, 50, 100, 165, and 198 *equiv.* of aniline relative to the amount of Tp, respectively.

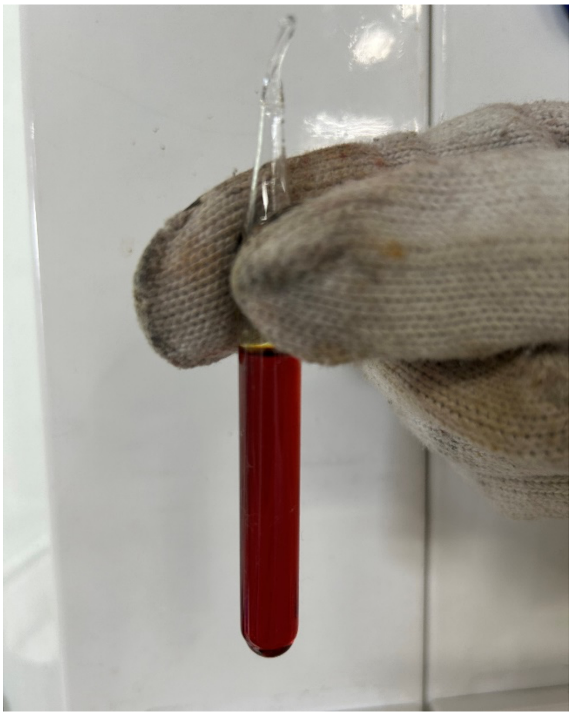

Supplementary Fig. 7 Photograph of the reaction solution.

Photograph of the post-reaction solution using excessive aniline (300 *equiv.*) for synthesizing TpPa-SCOF-An.

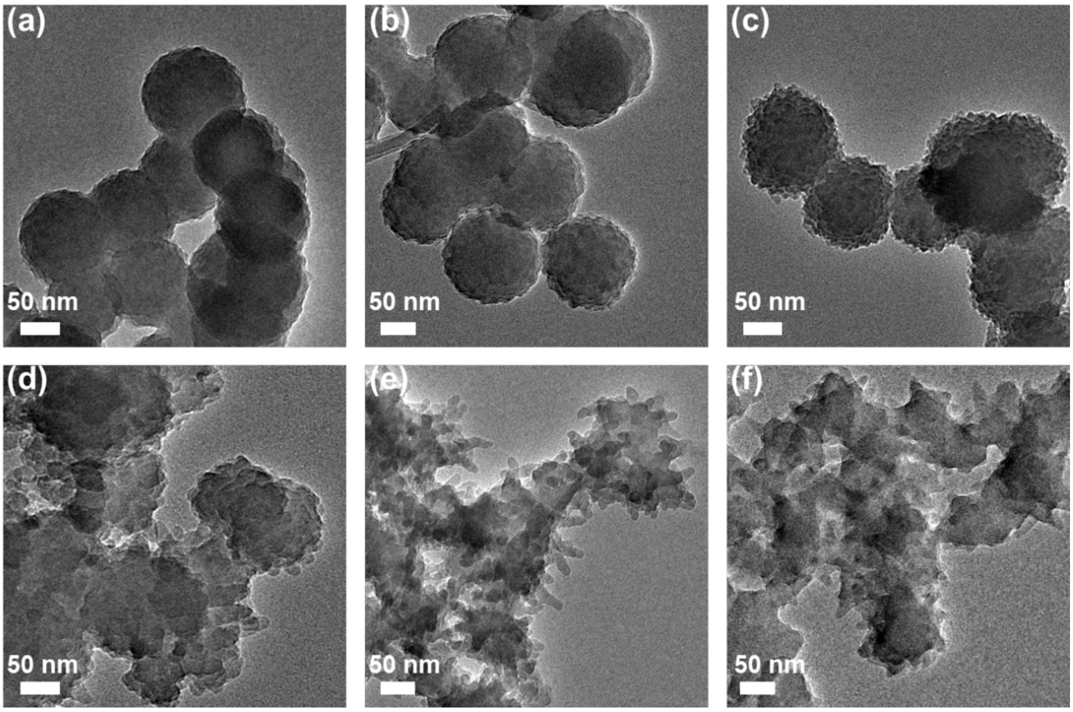

Supplementary Fig. 8 TEM images for TpPa-SCOF-An synthesized under different conditions.

TEM images of TpPa-SCOF-An prepared with (a) 8 *equiv.*, (b) 25 *equiv.*, (c) 50 *equiv.*, (d) 100 *equiv.*, (e) 165 *equiv.*, and (f) 200 *equiv.* of aniline, respectively.

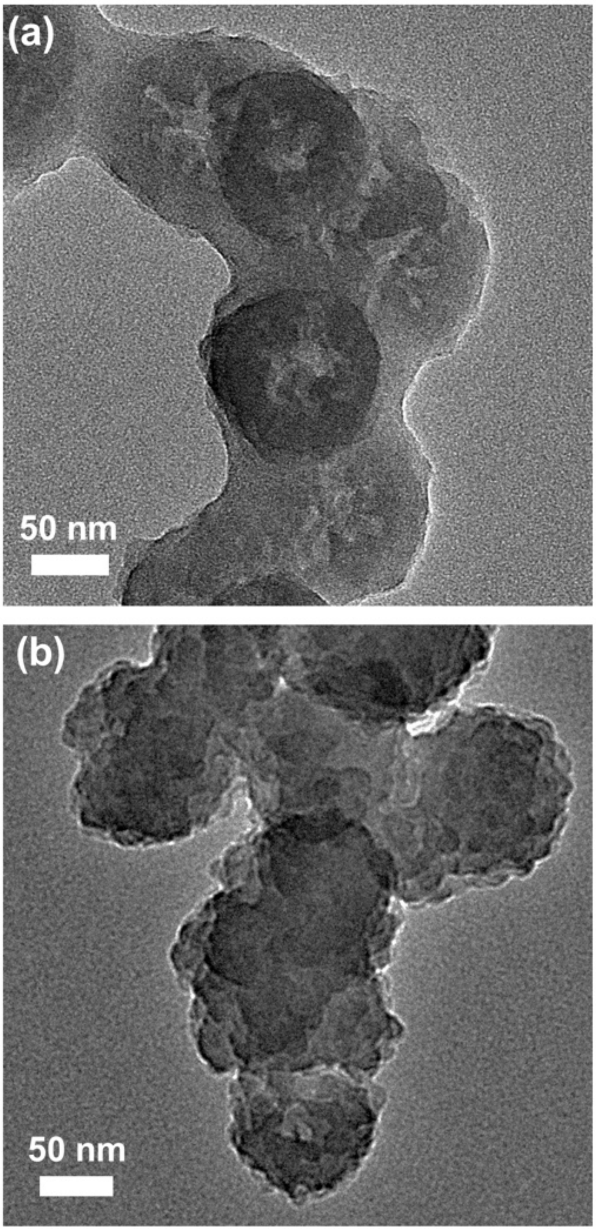

Supplementary Fig. 9 TEM images of the spherical COFs after etching with alkali. TEM images of (a) TpPa-SCOF-An and (b) TpPa-SCOF exposed to 3 M NaOH aqueous solution for 24 hours. The recovered solids were further rinsed with water and THF several times. One can see that TpPa-SCOF-An appears internal cavities or slits with smooth surface, while TpPa-SCOF is changed to be accumulation of small grains with solid insides.

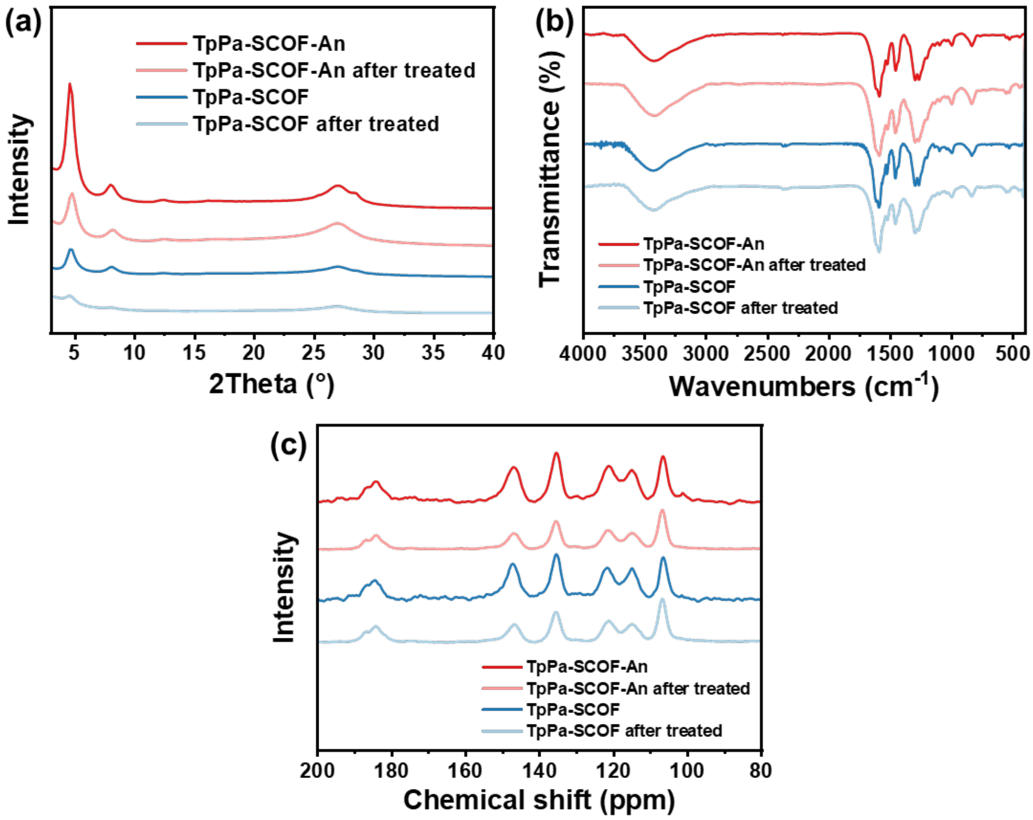

Supplementary Fig. 10 Characterizations of the spherical COFs after etching.

(a) PXRD patterns, (b) FT IR spectra, and (c) solid-state CP/MAS ^{13}C NMR spectra of TpPa-SCOF-An and TpPa-SCOF before and after being exposed to 3M NaOH aqueous solution for 24 h.

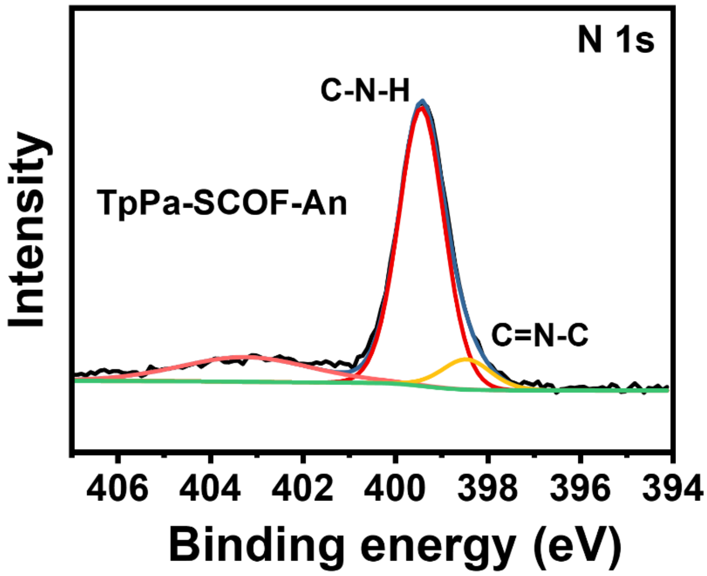

Supplementary Fig. 11 XPS spectrum.

The XPS N1*s* spectrum of TpPa-SCOF-An.

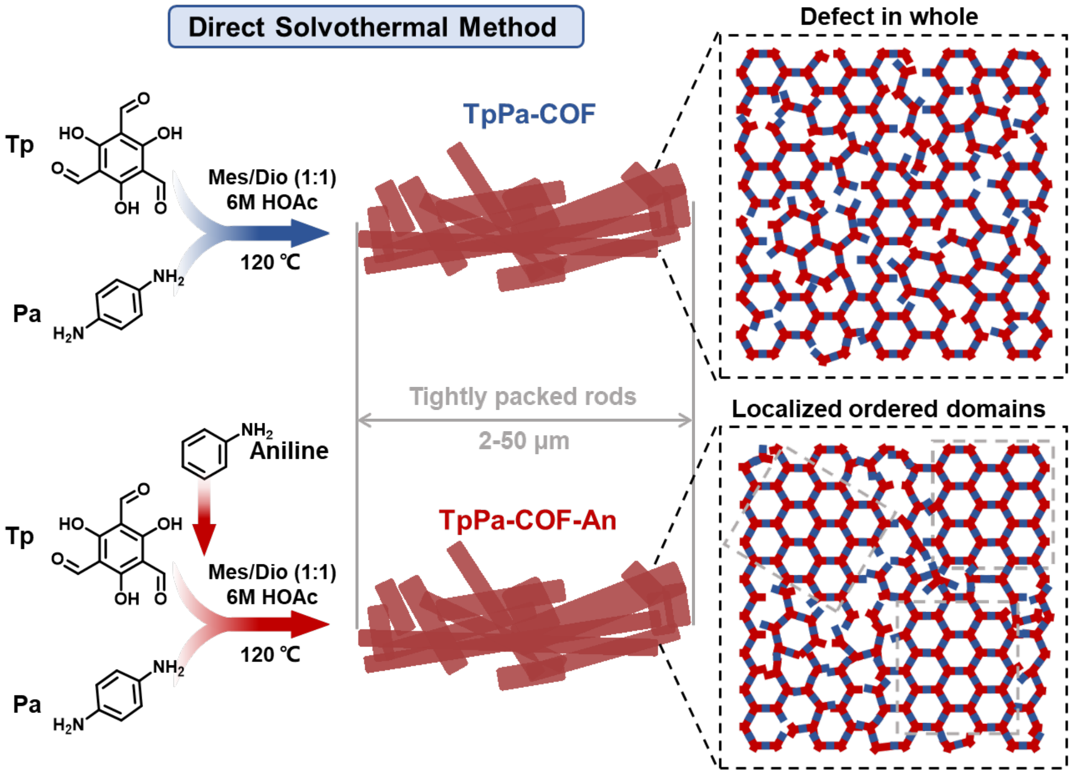

Supplementary Fig. 12 Illustration of the solvothermal synthesis of TpPa-COF. Preparation of TpPa-COF (top) and TpPa-COF-An (bottom) through the direct solvothermal method and regulator-induced solvothermal method, respectively.

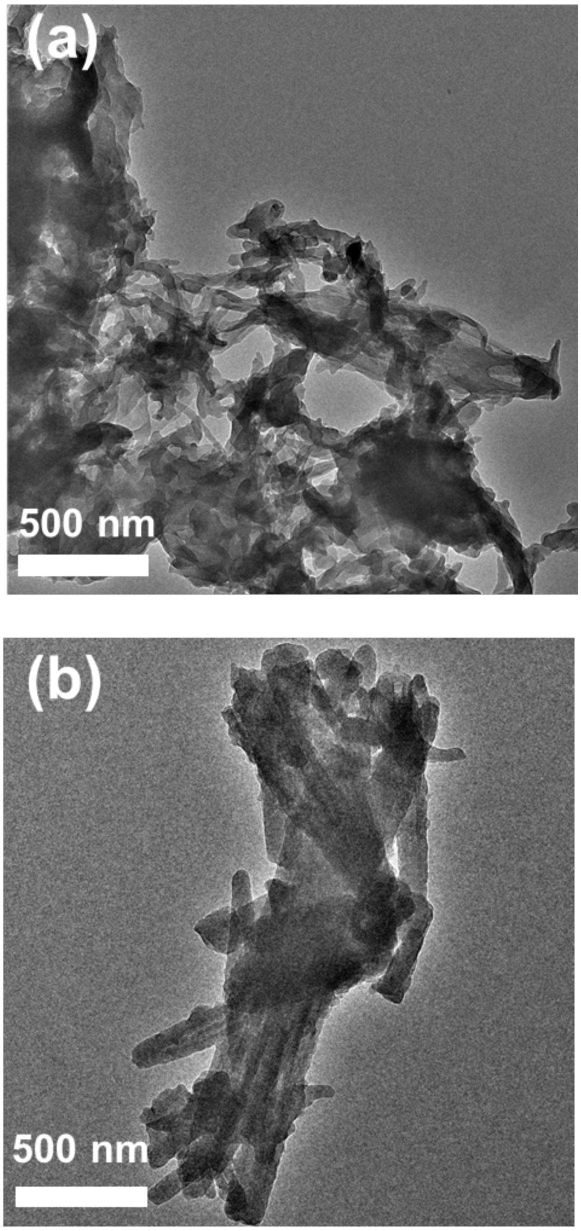

Supplementary Fig. 13 TEM images of COFs.

TEM images of (a) TpPa-COF-An and (b) TpPa-COF.

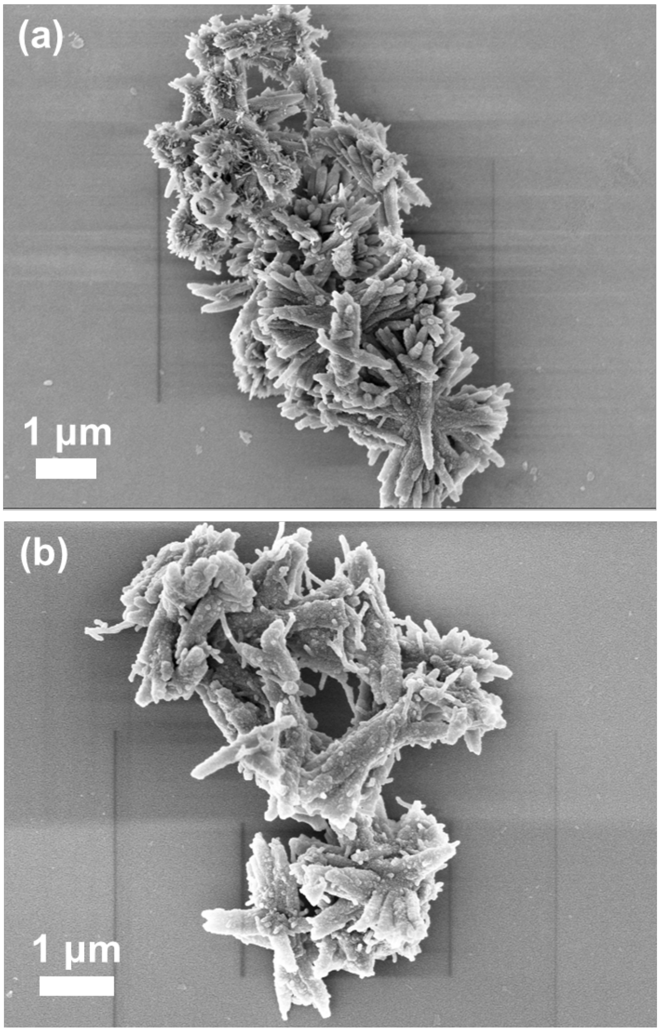

Supplementary Fig. 14 SEM images of COFs.

SEM images of (a) TpPa-COF-An and (b) TpPa-COF.

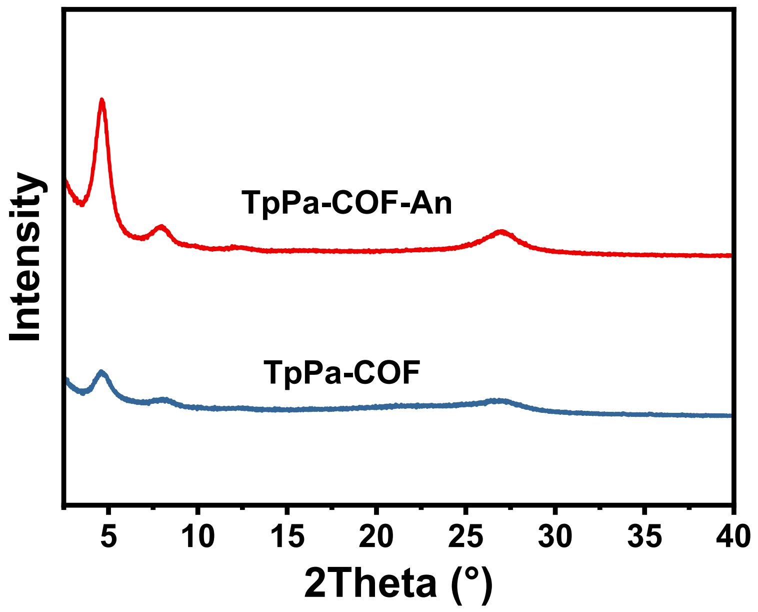

Supplementary Fig. 15 Structural characterization of COFs.

PXRD patterns of TpPa-COF-An and TpPa-COF.

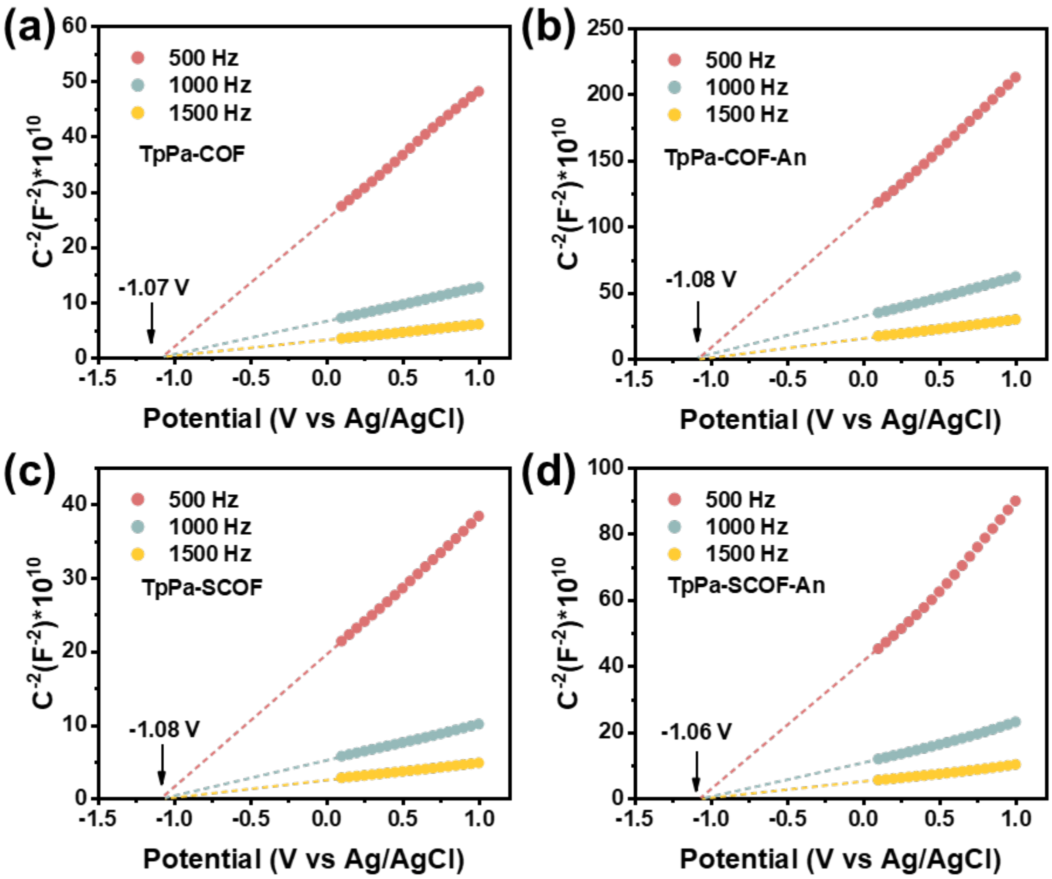

Supplementary Fig. 16 Mott-Schottky plots of COFs.

Mott-Schottky plots of (a) TpPa-COF, (b) TpPa-COF-An, (c) TpPa-SCOF, and (d) TpPa-SCOF-An, respectively.

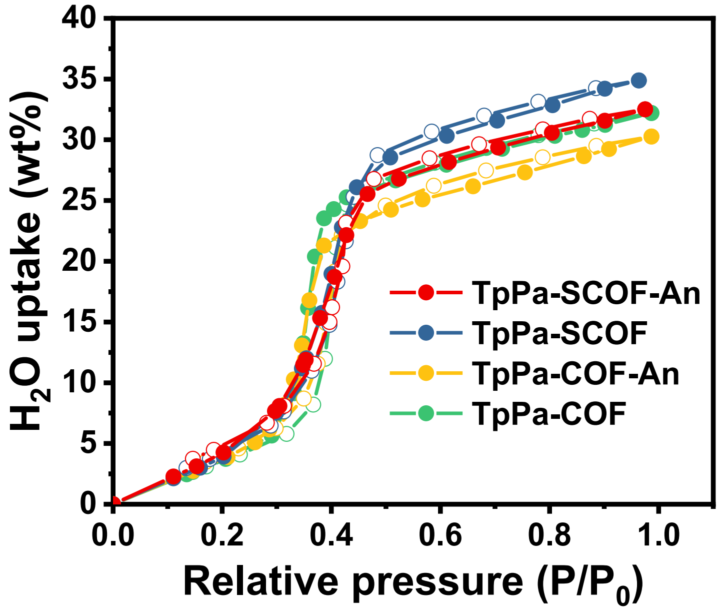

Supplementary Fig. 17 Water sorption isotherms of COFs.

Water adsorption isotherms (filled symbols) and desorption isotherms (open symbols) for TpPa-SCOF-An, TpPa-SCOF, TpPa-COF-An, and TpPa-COF. The water adsorption capacities were 32.5, 34.8, 30.2, and 32.1*wt*%, respectively.

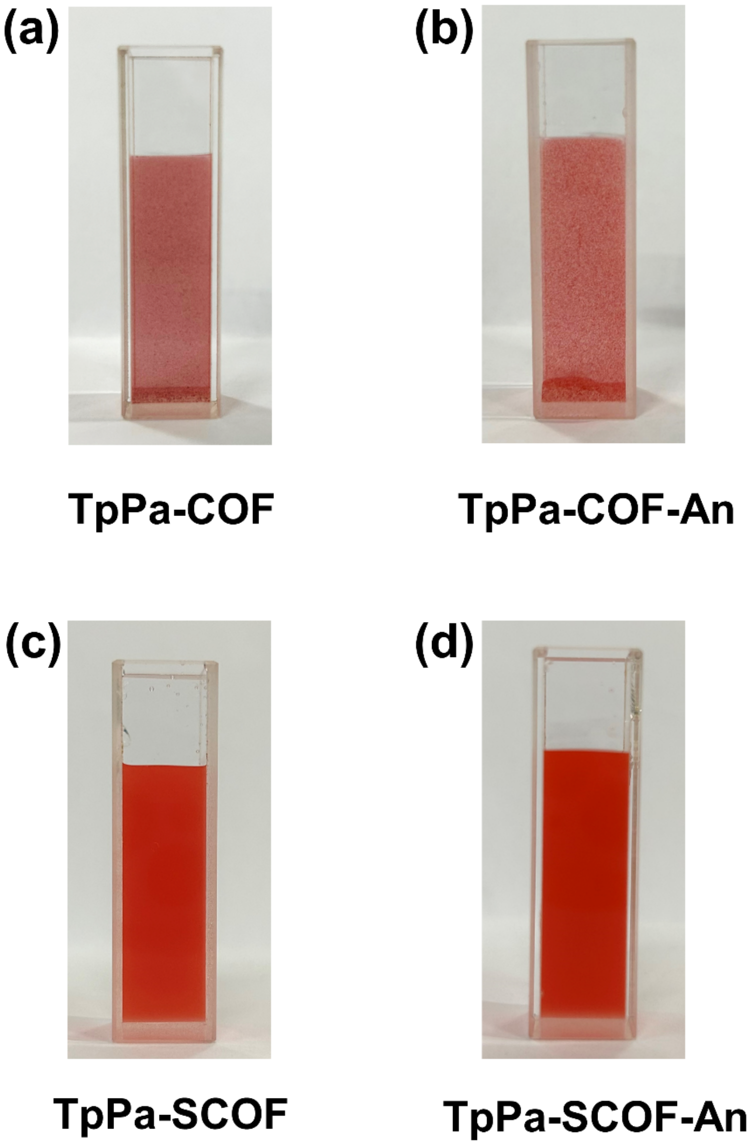

Supplementary Fig. 18 Photographs of the COF dispersions.

Photographs of the aqueous dispersions (0.1 mg mL^{-1}) of (a) TpPa-COF, (b) TpPa-COF-An, (c) TpPa-SCOF, and (d) TpPa-SCOF-An.

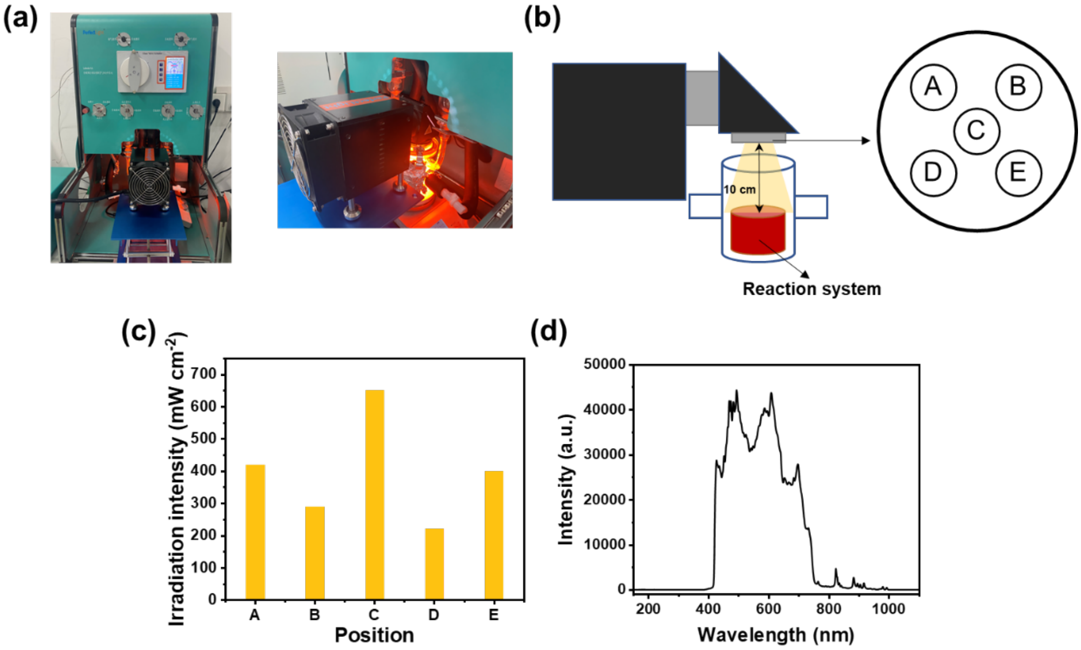

Supplementary Fig. 19 Light source conditions for photocatalysis.

(a) Photograph of the device used in the photocatalytic H_2 evolution tests. (b) Scheme of the light irradiation condition. (c) The irradiation intensity measured for the position illustrated in Scheme b. (d) Light source spectra for the 300 W Xe lamp equipped with a cut-off filter (>420 nm).

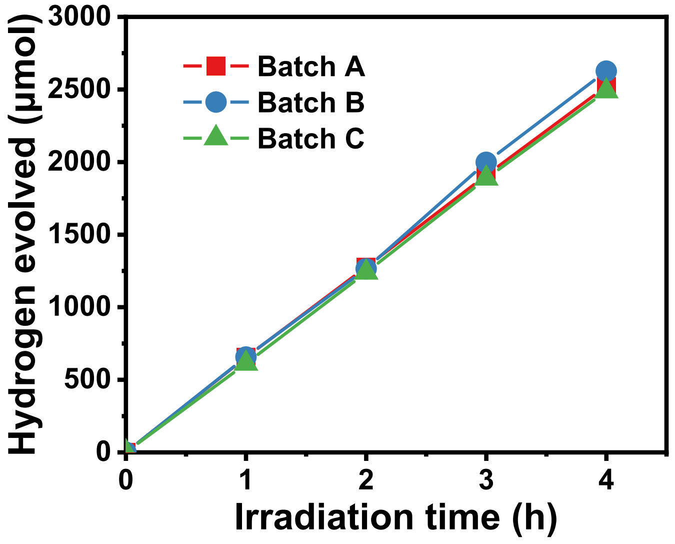

Supplementary Fig. 20 Repetitive test for photocatalytic H_2 evolution.

Time course for photocatalytic H_2 production using TpPa-SCOF-An from three different batches.

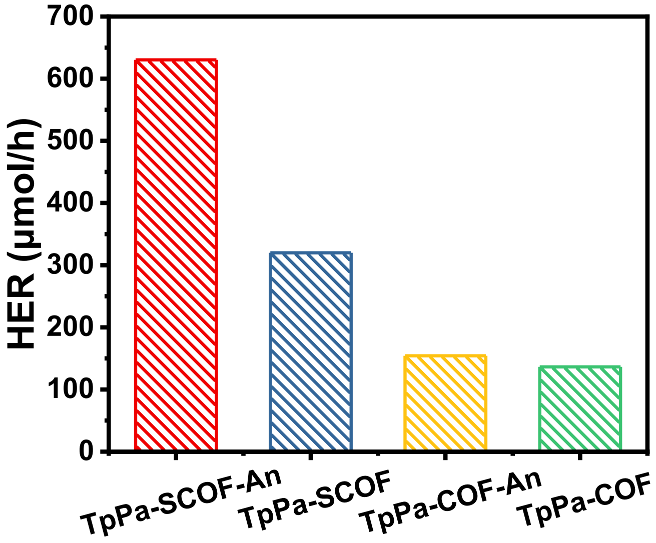

Supplementary Fig. 21 Photocatalytic H_2 evolution rates of COFs.

H_2 production rates for the different photocatalysts loaded with the similar Pt amount measured by ICP, which were 0.14, 0.19, 0.16, and 0.12 wt%, respectively, for TpPa-SCOF-An-Pt, TpPa-SCOF-Pt, TpPa-COF-An-Pt, and TpPa-COF-Pt. The results suggest that the observed difference in photocatalytic activity is not due to variation in the exact Pt loading amounts.

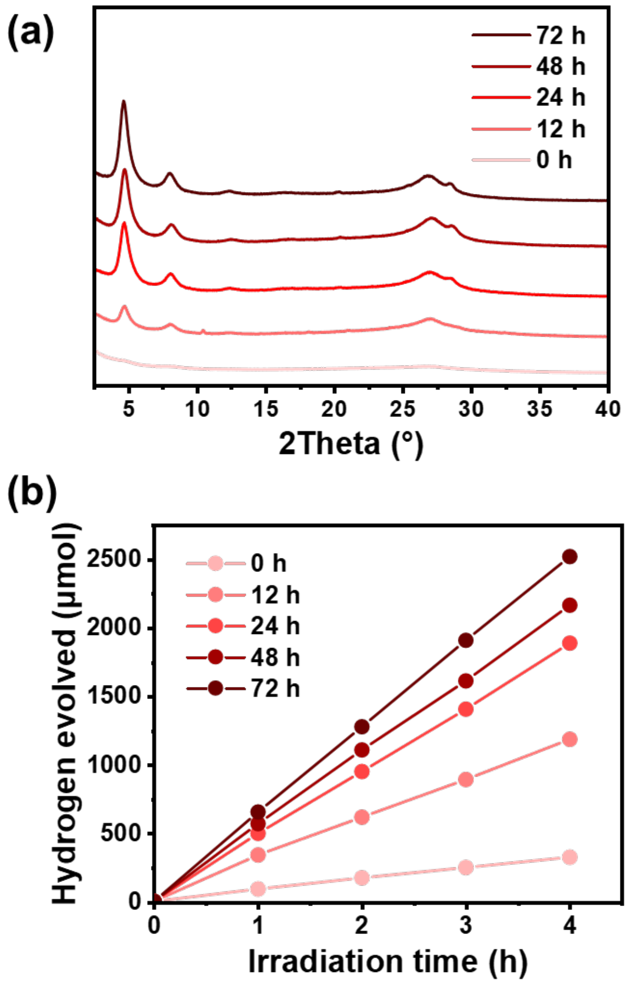

Supplementary Fig. 22 Correlation between COF crystallinity and photocatalytic performance.

(a) PXRD patterns and (b) time-dependent hydrogen evolution curves for TpPa-SCOF-An synthesized by prolonging solvothermal reaction time.

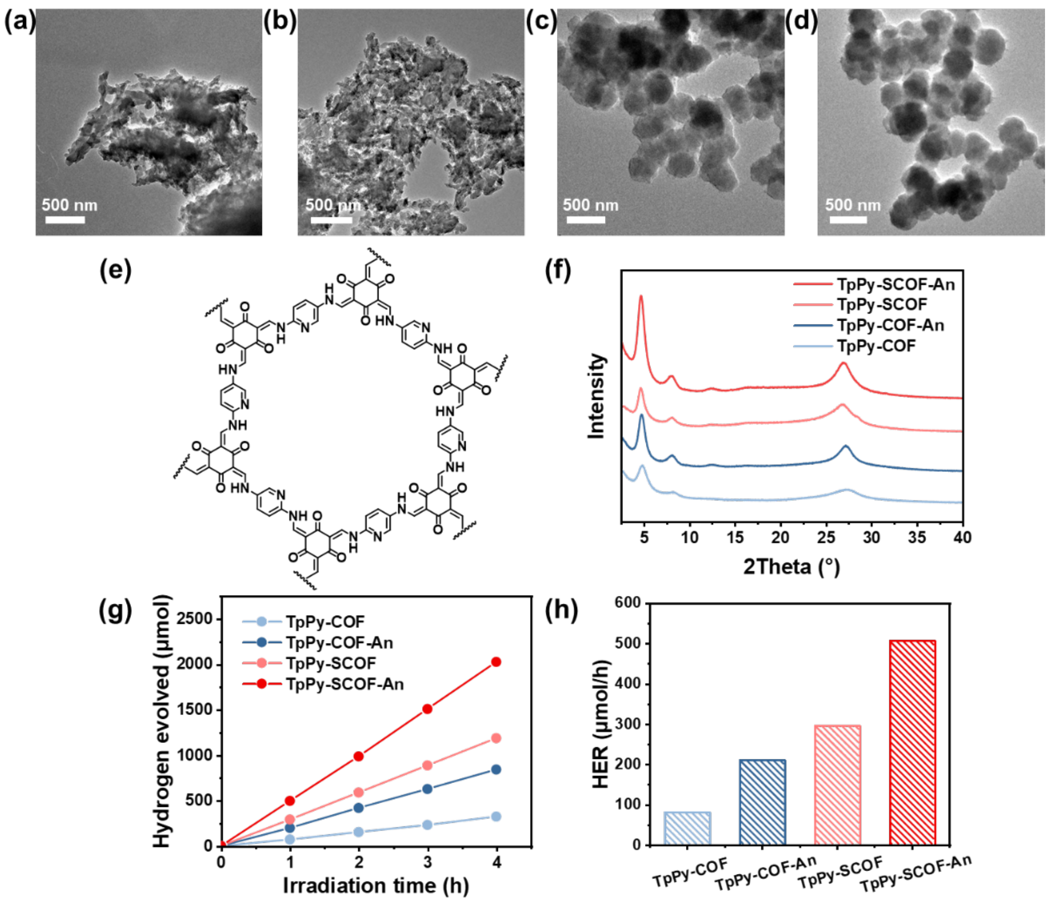

Supplementary Fig. 23 Morphological, structural and photocatalytic performance characterizations for TpPy-COF.

TEM images for (a) TpPy-COF, (b) TpPy-COF-An, (c) TpPy-SCOF, and (d) TpPy-SCOF-An. (e) Chemical structure of TpPy-COF. (f) PXRD patterns, (g) time course for photocatalytic H_2 production under visible light irradiation, and (h) average H_2 production rates for different photocatalysts. The average diameters of TpPy-SCOF and TpPy-SCOF-An were ~400 nm. The HER values were 87, 211, 297, and 507 μmol h^{-1}, respectively.

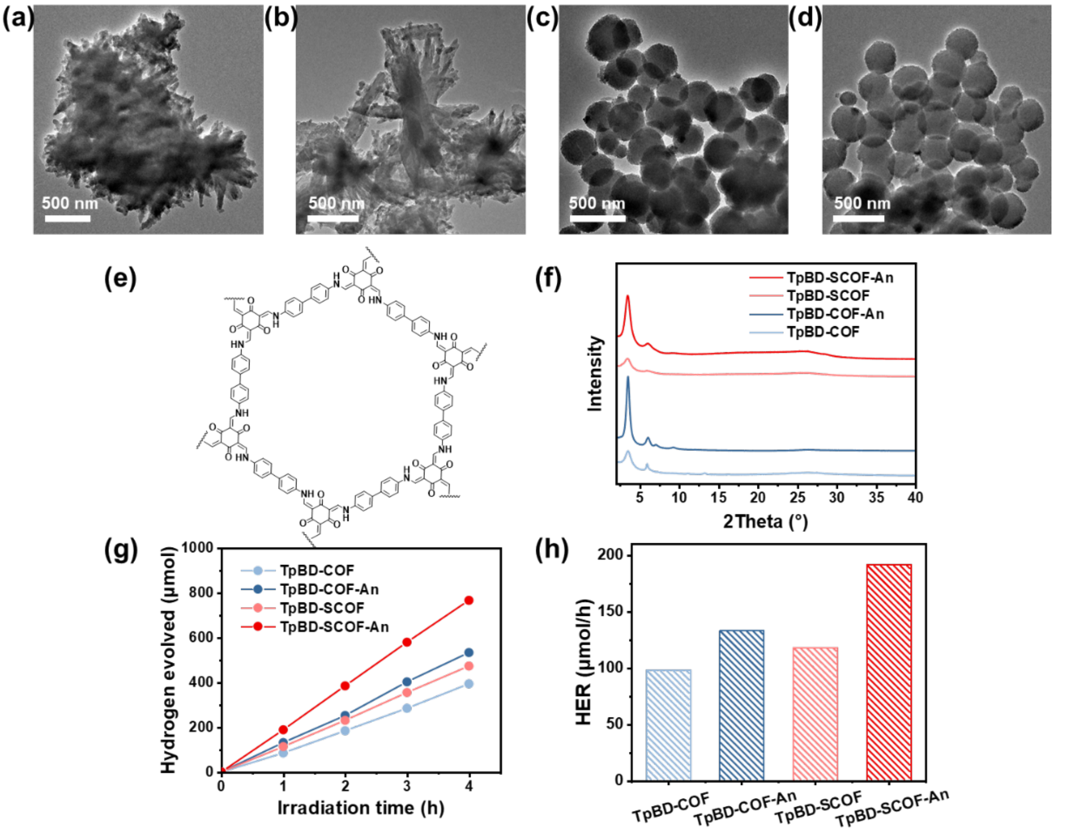

Supplementary Fig. 24 Morphological, structural and photocatalytic performance characterizations for TpBD-COF.

TEM images for (a) TpBD-COF, (b) TpBD-COF-An, (c) TpBD-SCOF, and (d) TpBD-SCOF-An. (e) Chemical structure of TpBD-COF. (f) PXRD patterns, (g) time course for photocatalytic H_2 production under visible light irradiation, and (h) average H_2 production rates for different photocatalysts. The average diameters of TpBD-SCOF and TpBD-SCOF-An were ~300 nm. The HER values were 98, 133, 120, and 192 μmol h^{-1}, respectively.

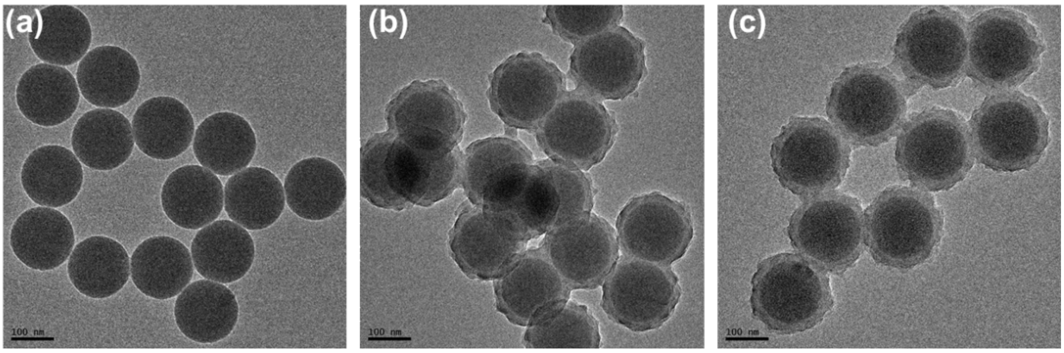

Supplementary Fig. 25 Morphological characterization for SiO_2@COF.

TEM images for (a) SiO_2, (b) SiO_2@TpPa-Polymer, and (c) SiO_2@TpPa-An, respectively.

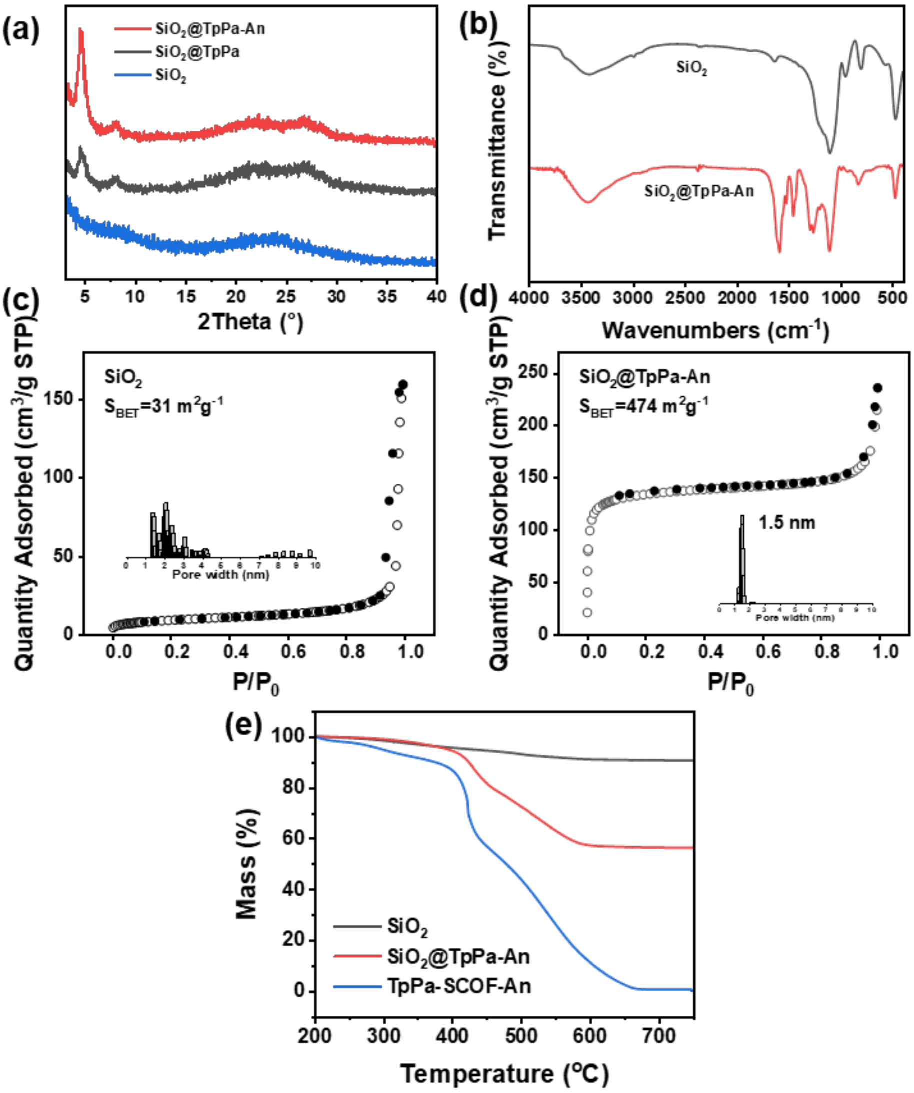

Supplementary Fig. 26 Characterizations for SiO_2@COF.

(a) PXRD patterns of SiO_2@TpPa-An, SiO_2@TpPa, and SiO_2. (b) FT IR spectra of SiO_2 and SiO_2@TpPa-An. (c,d) Nitrogen adsorption (open circle) and desorption (solid circle) isotherm profiles for (c) SiO_2 and (d) SiO_2@TpPa-An. (e) TGA curves of SiO_2, SiO_2@TpPa-An, and TpPa-SCOF-An. After 700°C, all organic components were decomposed completely. Thus, compared to SiO_2 and TpPa-SCOF-An, the weight percent of TpPa- An shell in SiO_2@TpPa-An was calculated to be 37wt%.

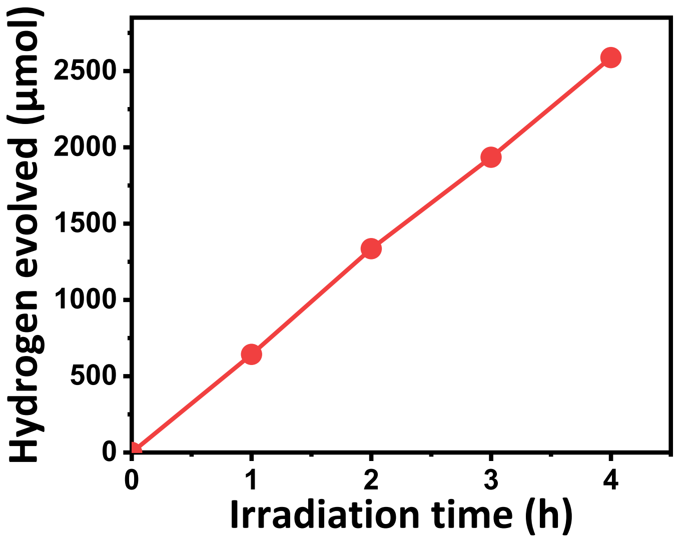

Supplementary Fig. 27 Photocatalytic performance of SiO_2@COF.

Time course for photocatalytic H_2 production under visible irradiation for SiO_2@TpPa-An.

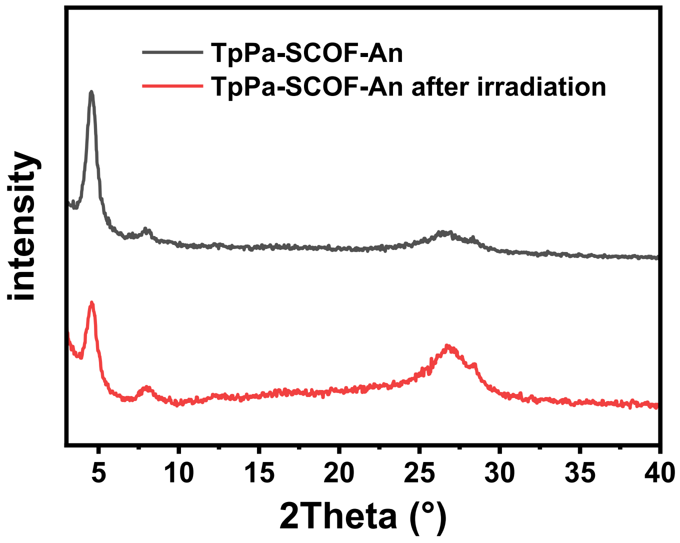

Supplementary Fig. 28 Structural characterization for the recycled COF.

PXRD patterns of TpPa-SCOF-An before and after irradiation.

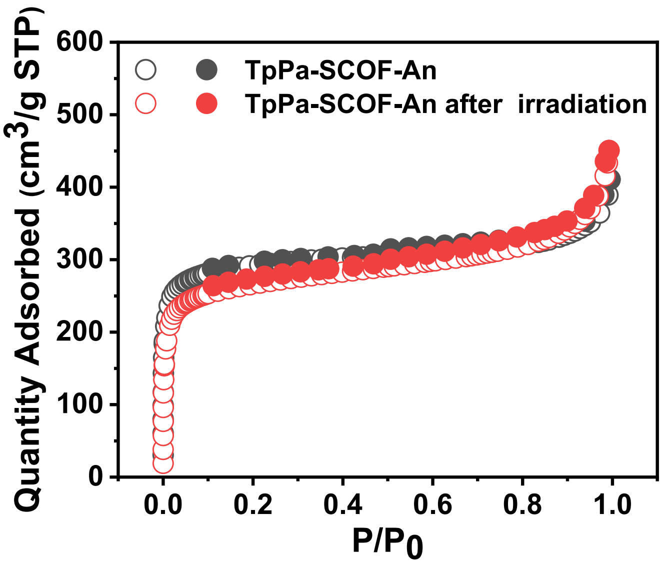

Supplementary Fig. 29 Porous characterization for the recycled COF.

Nitrogen adsorption (open circle) and desorption (solid circle) isotherm profiles of TpPa-SCOF-An before and after irradiation.

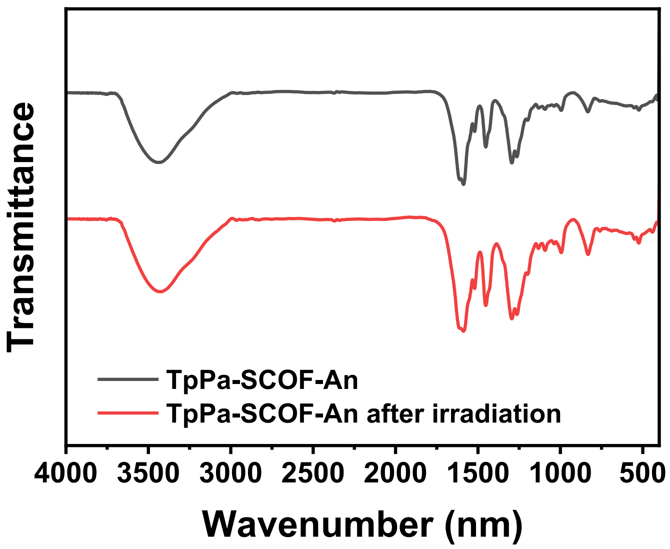

Supplementary Fig. 30 Composition characterization for the recycled COF.

FT IR spectra of TpPa-SCOF-An before and after irradiation.

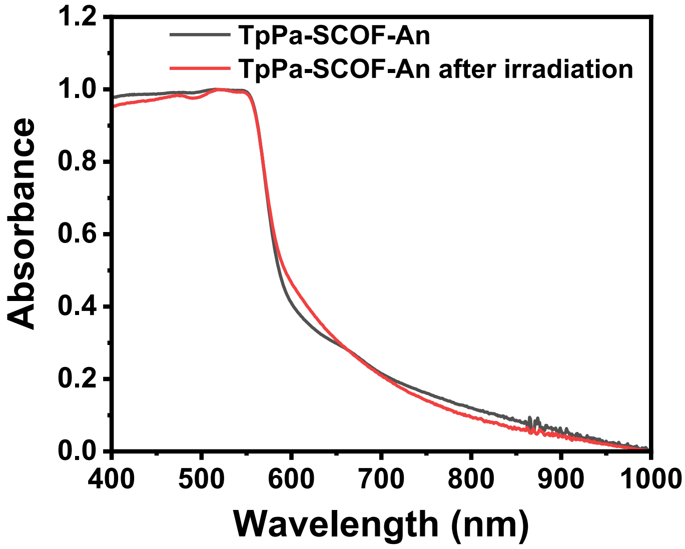

Supplementary Fig. 31 Absorption property of the recycled COF.

UV-vis diffuse reflectance spectra of TpPa-SCOF-An before and after irradiation.

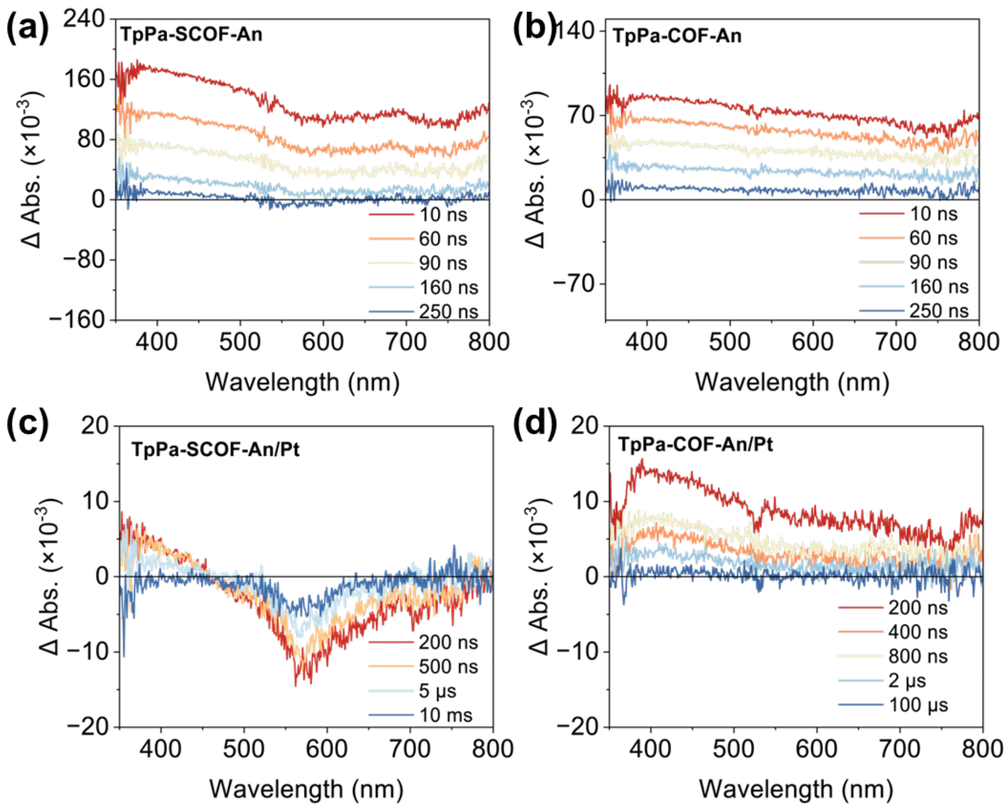

Supplementary Fig. 32 Transient absorption property of COFs.

TA spectra of **(a)** TpPa-SCOF-An, (b) TpPa-COF-An, (c) TpPa-SCOF-An/Pt and (d) TpPa-COF-An/Pt suspension in aqueous solution under inert atmosphere at different time delay after pulsed laser excitation (λ_{ex} = 532 nm, 5 mJ/pulse).

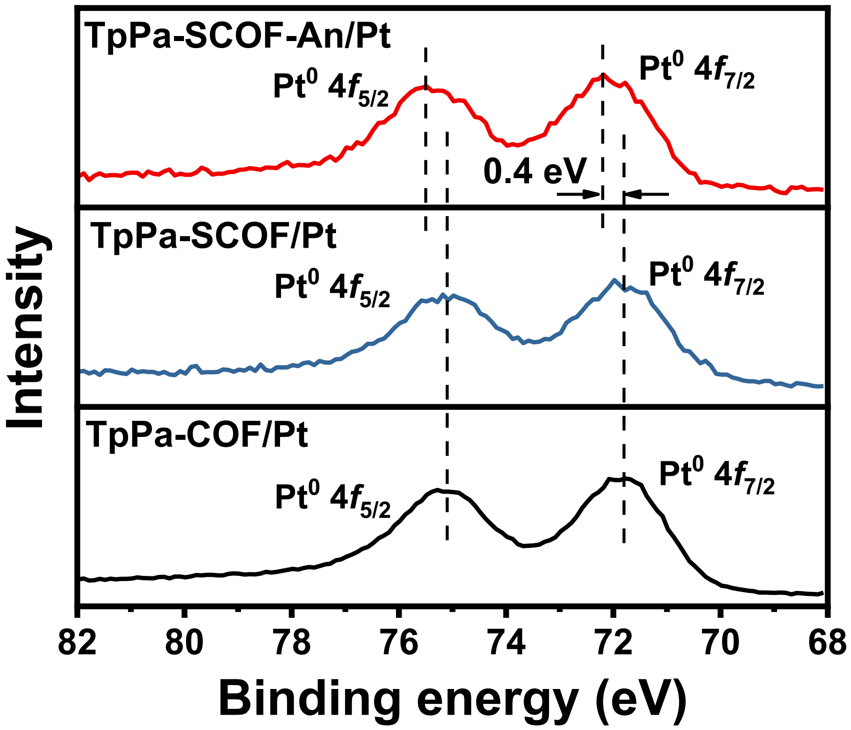

Supplementary Fig. 33 XPS spectra of Pt-loaded COFs.

High-resolution XPS spectra of Pt 4*f* for TpPa-SCOF-An/Pt, TpPa-SCOF/Pt, and TpPa-COF/Pt.

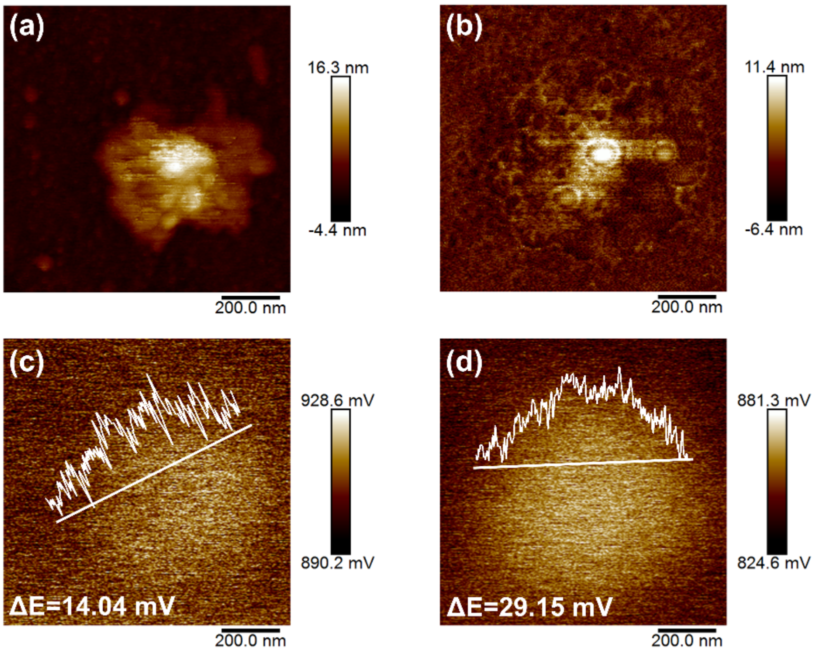

Supplementary Fig. 34 Kelvin Probe Force Microscopy characterization for the spherical COFs.

(a) The height image and (c) the corresponding surface potential image of TpPa-SCOF. (b) The height image and (d) the corresponding surface potential image of TpPa-SCOF-An.

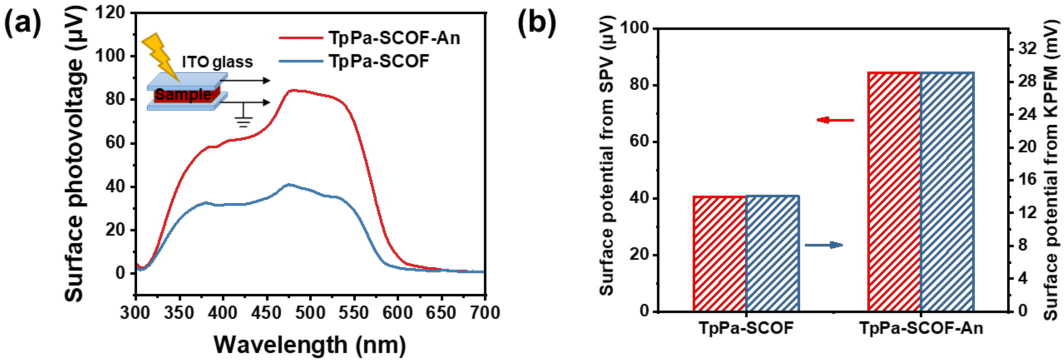

Supplementary Fig. 35 Surface photovoltage characterization for the spherical COFs.

(a) Surface photovoltage for TpPa-SCOF-An and TpPa-SCOF and (b) the comparison of surface potential derived from SPV and KPFM, respectively.

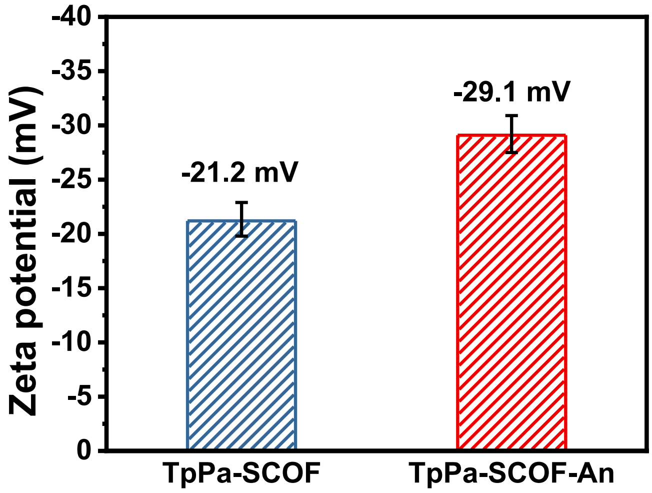

Supplementary Fig. 36 Zeta potential comparison of COFs.

Zeta potentials of TpPa-SCOF-An and TpPa-SCOF. The experiment was measured at a concentration of 0.1 g/L samples with 0.01 M NaCl and at 25°C. Error bars indicate the error in the measurement.

Supplementary Table 1. Yields of TpPa-SCOF-An synthesized with different added amounts of aniline under otherwise identical conditions.

**Amounts of aniline (*equiv.*)**	**Yield**
1	91.5%
4	91.2%
8	90.0%
16	88.6%
25	80.4%
50	74.4%
100	59.6%
165	46.2%
200	21.5%
300	0

Supplementary Table 2. Comparison of photocatalytic H_2 evolution performances with different COF-based photocatalysts.

Catalyst	Co-catalyst	Sacrificial agent	HER (μmol $g^{-1}h^{-1}$)	AQE (%)	Ref.
**TpPa-SCOF-An**	**Pt**	**Ascorbic acid**	**126,000**	**15.96 (475 nm)**	**This work**
**SiO_2@TpPa-An**	**Pt**	**Ascorbic acid**	**350,000**		**This work**
NH_2-UiO-66/TpPa-1-COF(4 : 6)	Pt	Sodium ascorbate	23,413	-	[12]
FS-COF + WS5F	Pt	Ascorbic acid	16,300	2.2 (600 nm)	[13]
ATNT-4	Pt	Ascorbic acid	14,228.1	9.75 (500 nm)	[14]
Pd^0/TpPa-1-EosinY	Pd	TEOA	10,400	3.2 (600 nm)	[15]
CN-COF	Pt	TEOA	10,100	20.7 (425 nm)	[16]
Pt-PVP-TP-COF	Pt	Ascorbic acid	8,420	0.4 (475 nm)	[17]
COF-JLU100	Pt	Ascorbic acid	107,380	5.13 (450 nm)	[18]
Tp-2C/BPy2+-COF (19.10%)	Pt	Ascorbic acid	34,600	6.93 (420 nm)	[19]
TtaTfa	Pt	Ascorbic acid	20,700	1.43 (450 nm)	[20]
$Ni(OH)_2$-2.5%/TpPa-2	$Ni(OH)_2$	Sodium ascorbate	1,896	-	[21]
ZnPor-DETH-COF	Pt	TEOA	413	0.063 (420 nm)	[22]
TCDA-COF (COF-JLU35)	Pt	Ascorbic acid	70,800	2.57 (450 nm)	[23]
PY-DHBD-COF	Pt	Ascorbic acid	71,160	8.4% (420 nm)	[24]

TiO_2-TpPa-1-COF	Pt	Sodium ascorbate	11,190	7.6 (420 nm)	[25]
Mo_3S_{13}@EB-COF	Ru	Ascorbic acid	13,215	4.49 (475 nm)	[26]
Ni-Py-COF	Pt	Ascorbic acid	13,231	-	[27]
RC-COF-1	Pt	Ascorbic acid	27,980	6.39 (420 nm)	[28]
TpBpy-Ni2%	Pt	Ascorbic acid	51,300	5.33 (475 nm)	[29]
CYANO-CON	Pt	Ascorbic acid	134,200	82.6 (450 nm)	[30]
USTB-10	Pt	Ascorbic acid	21,800	0.68 (420 nm)	[31]
COF-954	Pt	Ascorbic acid	137,230	5.00 (550 nm)	[32]

Supplementary Table 3. The COF loading content of SiO_2@TpPa-An microspheres derived from ICP-AES analysis.

Sample	Si content (wt%)	COF content (wt%)
SiO_2	12.73	/
SiO_2@TpPa-An	8.14	36.1

Supplementary Table 4. Fitting Parameters Based on the Multi-exponential Function.

Sample	A_1	t_1 (s)	A_2	t_2 (s)	A_3	t_3 (s)	$\bar{t}$ (s)[a]
TpPa-SCOF-An/Pt	0.783	4.82E-07	0.330	5.66E-06	0.0852	1.19E-04	9.85E-05
TpPa-SCOF-An	74.9	2.36E-08	-	-	-	-	-
TpPa-COF-An/Pt	0.991	7.81E-08	-	-	-	-	-
TpPa-COF-An	2.64	1.06E-07	-	-	-	-	-

[a] The average lifetime is calculated based on the following equation,

$$\bar{t} = \frac{A_1 t_1^2 + A_2 t_2^2 + A_3 t_3^2}{A_1 t_1 + A_2 t_2 + A_3 t_3}$$

Supplementary Table 5. Overpotentials of photocatalysts before and after Pt deposition derived from linear sweep voltammetry.

Sample	Overpotential (V *vs.* RHE)	
	Before Pt deposition	After Pt deposition
TpPa-SCOF-An	-1.298	-1.201
TpPa-COF-An	-1.308	-1.248
TpPa-SCOF	-1.322	-1.272
TpPa-COF	-1.325	-1.288

Supplementary References

[1] Kong, W. *et al.* Amorphous-to-crystalline transformation toward controllable synthesis of fibrous covalent organic frameworks enabling promotion of proton transport. *Chem. Commun.* **55**, 75-78, doi:10.1039/C8CC08590K (2019).

[2] Wang, R., Kong, W., Zhou, T., Wang, C. & Guo, J. Organobase modulated synthesis of high-quality β-ketoenamine-linked covalent organic frameworks. *Chem. Commun.* **57**, 331-334, doi:10.1039/D0CC06519F (2021).

[3] Zhu, Y.-L. *et al.* Building block design for minimizing defects in the construction of two-dimensional covalent organic frameworks. *J. Phys. Chem. Lett.* **11**, 179-183, doi:10.1021/acs.jpclett.9b03420 (2019).

[4] Yu, X.-K. *et al.* Mechanism for topology selection of isomeric two-dimensional covalent organic frameworks. *J. Phys. Chem. Lett.* **13**, 7087-7093, doi: 10.1021/acs.jpclett.2c01743 (2022).

[5] Zhu, Y.-L. *et al.* GALAMOST: GPU-accelerated large-scale molecular simulation toolkit. *J. Comput. Chem.* **34**, 2197-2211, doi:10.1002/jcc.23365 (2013).

[6] Zhu, Y.-L. *et al.* Employing multi-GPU power for molecular dynamics simulation: an extension of GALAMOST. *Mol. Phys.* **116**, 1065-1077, doi:10.1080/00268976.2018.1434904 (2018).

[7] Anantharaj, S. *et al.* Precision and correctness in the evaluation of electrocatalytic water splitting: revisiting activity parameters with a critical assessment. *Energy Environ. Sci.* **11**, 744-771, doi:10.1039/C7EE03457A (2018).

[8] Seo Im, J. *et al.* Reduction of oscillator strength due to piezoelectric fields in GaN/$Al_xGa_{1-x}N$ quantum wells. *Phys. Rev. B* **57**, R9435-R9438, doi:10.1103/PhysRevB.57.R9435 (1998).

[9] Lefebvre, P. *et al.* Time-resolved photoluminescence as a probe of internal electric fields in GaN-(GaAl)N quantum wells. *Phys. Rev. B* **59**, 15363-15367, doi:10.1103/PhysRevB.59.15363 (1999).

[10] Morello, G. *et al.* Intrinsic optical nonlinearity in colloidal seeded grown CdSe/CdS nanostructures: Photoinduced screening of the internal electric field. *Phys.*

Rev. B **78**, 195313, doi:10.1103/PhysRevB.78.195313 (2008).

[11] Li, J., Zhan, G., Yu, Y. & Zhang, L. Superior visible light hydrogen evolution of Janus bilayer junctions via atomic-level charge flow steering. *Nat. Commun.* **7**, 11480, doi:10.1038/ncomms11480 (2016).

[12] Zhang, F.-M. *et al.* Rational Design of MOF/COF Hybrid Materials for Photocatalytic H2 Evolution in the Presence of Sacrificial Electron Donors. *Angew. Chem. Int. Ed.* **57**, 12106-12110, doi:10.1002/anie.201806862 (2018).

[13] Wang, X. *et al.* Sulfone-containing covalent organic frameworks for photocatalytic hydrogen evolution from water. *Nat. Chem.* **10**, 1180-1189, doi:10.1038/s41557-018-0141-5 (2018).

[14] Wang, H. *et al.* Integrating Suitable Linkage of Covalent Organic Frameworks into Covalently Bridged Inorganic/Organic Hybrids toward Efficient Photocatalysis. *J. Am. Chem. Soc.* **142**, 4862-4871, doi:10.1021/jacs.0c00054 (2020).

[15] Ding, S.-Y., Wang, P.-L., Yin, G.-L., Zhang, X. & Lu, G. Energy transfer in covalent organic frameworks for visible-light-induced hydrogen evolution. *Int. J. Hydrogen Energ.* **44**, 11872-11876, doi:10.1016/j.ijhydene.2019.03.039 (2019).

[16] Luo, M. L., Yang, Q., Liu, K. W., Cao, H. M. & Yan, H. J. Boosting photocatalytic H_2 evolution on *g*-C_3N_4 by modifying covalent organic frameworks (COFs). *Chem. Commun.* **55**, 5829-5832, doi:10.1039/c9cc02144b (2019).

[17] Ming, J. *et al.* Hot pi-Electron Tunneling of Metal-Insulator-COF Nanostructures for Efficient Hydrogen Production. *Angew. Chem. Int. Ed.* **58**, 18290-18294, doi:10.1002/anie.201912344 (2019).

[18] Ma, S. *et al.* Photocatalytic Hydrogen Production on a sp^2 Carbon Linked Covalent Organic Framework. *Angew. Chem. Int. Ed.* **61**, e202208919, doi:10.1002/anie.202208919 (2022).

[19] Mi, Z. *et al.* Covalent Organic Frameworks Enabling Site Isolation of Viologen-Derived Electron-Transfer Mediators for Stable Photocatalytic Hydrogen Evolution. *Angew. Chem. Int. Ed.* **60**, 9642-9649, doi:10.1002/anie.202016618 (2021).

[20] Yang, J. *et al.* Protonated Imine-Linked Covalent Organic Frameworks for Photocatalytic Hydrogen Evolution. *Angew. Chem. Int. Ed.* **60**, 19797-19803,

doi:10.1002/anie.202104870 (2021).
[21] Dong, H. *et al.* Boosting visible-light hydrogen evolution of covalent-organic frameworks by introducing Ni-based noble metal-free co-catalyst. *Chem. Eng. J.* **379**, 122342, doi:10.1016/j.cej.2019.122342 (2020).
[22] Chen, R. *et al.* Rational design of isostructural 2D porphyrin-based covalent organic frameworks for tunable photocatalytic hydrogen evolution. *Nat. Commun.* **12**, 1354, doi:10.1038/s41467-021-21527-3 (2021).
[23] Li, Z. *et al.* Three-Component Donor−π–Acceptor Covalent–Organic Frameworks for Boosting Photocatalytic Hydrogen Evolution. *J. Am. Chem. Soc.* **145**, 8364–8374, doi:10.1021/jacs.2c11893 (2023).
[24] Li, Y. *et al.* In situ photodeposition of platinum clusters on a covalent organic framework for photocatalytic hydrogen production. *Nat. Commun.* **13**, 1355, doi:10.1038/s41467-022-29076-z (2022).
[25] Li, C.-C. *et al.* Rational combination of covalent-organic framework and nano TiO2 by covalent bonds to realize dramatically enhanced photocatalytic activity. *Appl. Catal. B Environ.* **266**, 118586, doi: 10.1016/j.apcatb.2020.118586 (2020).
[26] Cheng, Y.-J. *et al.* Encapsulating $[Mo_3S_{13}]^{2-}$clusters in cationic covalent organic frameworks: enhancing stability and recyclability by converting a homogeneous photocatalyst to a heterogeneous photocatalyst. *Chem. Commun.* **54**, 13563-13566, doi:10.1039/C8CC07784C (2018).
[27] Sun, L. *et al.* Nickel Glyoximate Based Metal–Covalent Organic Frameworks for Efficient Photocatalytic Hydrogen Evolution. *Angew. Chem. Int. Ed.* **61**, e202204326, doi:10.1002/anie.202204326 (2022).
[28] Zhang, W. *et al.* Reconstructed covalent organic frameworks. *Nature* **604**, 72-79, doi:10.1038/s41586-022-04443-4 (2022).
[29] Zhang, H., Lin, Z., Kidkhunthod, P. & Guo, J. Stable Immobilization of Nickel Ions on Covalent Organic Frameworks for Panchromatic Photocatalytic Hydrogen Evolution. *Angew. Chem. Int. Ed.* **62**, e202217527, doi:10.1002/anie.202217527 (2023).
[30] Li, C., Liu, J., Li, H. *et al.* Covalent organic frameworks with high quantum efficiency in sacrificial photocatalytic hydrogen evolution. *Nat. Commun.* **13**, 2357,

doi:10.1038/s41467-022-30035-x (2022).

[31] Li, W., *et al*. Tuning Molecular Chromophores of Isoreticular Covalent Organic Frameworks for Visible Light-Induced Hydrogen Generation. *Adv. Funct. Mater.*, 32, 2207394, doi:10.1002/adfm.202207394 (2022)

[32] Zhong, Y., *et al*. Oligo(phenylenevinylene)-Based Covalent Organic Frameworks with Kagome Lattice for Boosting Photocatalytic Hydrogen Evolution. *Adv. Mater.*, 36, 2308251. doi:10.1002/adma.202308251 (2024)
